# Perioperative Intravenous Lidocaine and Metastatic Cancer Recurrence - A Narrative Review

**DOI:** 10.3389/fonc.2021.688896

**Published:** 2021-08-02

**Authors:** Thomas P. Wall, Donal J. Buggy

**Affiliations:** ^1^Department of Anaesthesiology, Mater Misericordiae University Hospital, School of Medicine, University College Dublin, Dublin, Ireland; ^2^EU COST Action 15204, Euro-Periscope, Brussels, Belgium; ^3^Outcomes Research, Cleveland Clinic, Cleveland, OH, United States

**Keywords:** cancer, recurrence, anaesthesia, surgery, local anaesthetics, lidocaine

## Abstract

Cancer is a major global health problem and the second leading cause of death worldwide. When detected early, surgery provides a potentially curative intervention for many solid organ tumours. Unfortunately, cancer frequently recurs postoperatively. Evidence from laboratory and retrospective clinical studies suggests that the choice of anaesthetic and analgesic agents used perioperatively may influence the activity of residual cancer cells and thus affect subsequent recurrence risk. The amide local anaesthetic lidocaine has a well-established role in perioperative therapeutics, whether used systemically as an analgesic agent or in the provision of regional anaesthesia. Under laboratory conditions, lidocaine has been shown to inhibit cancer cell behaviour and exerts beneficial effects on components of the inflammatory and immune responses which are known to affect cancer biology. These findings raise the possibility that lidocaine administered perioperatively as a safe and inexpensive intravenous infusion may provide significant benefits in terms of long term cancer outcomes. However, despite the volume of promising laboratory data, robust prospective clinical evidence supporting beneficial anti-cancer effects of perioperative lidocaine treatment is lacking, although trials are planned to address this. This review provides a state of the art summary of the current knowledge base and recent advances regarding perioperative lidocaine therapy, its biological effects and influence on postoperative cancer outcomes.

## Introduction

The burden of cancer as a global health issue is enormous – with an estimated 18.1 million new cases and 9.6 million related deaths in 2018, it is the second leading cause of death worldwide (1). Although the discovery of new chemotherapeutic agents and radiotherapy techniques continues to promise significant results, surgery is the mainstay of treatment for the majority of solid tumours that are detected prior to metastasis. Indeed, over 80% of all patients diagnosed with cancer will undergo a surgical procedure of some nature, for diagnostic or therapeutic purposes, with approximately 45 million surgical procedures estimated to be required per year by 2030 (2). Unfortunately, and despite optimal care, cancer often recurs following intended curative surgery in the form of metastatic disease. Metastatic cancer is typically refractory to treatment and is the most common cause of death in cancer patients (3). Therefore the importance of minimising recurrence risk is paramount. The physiological stress response induced by surgery stimulates inflammation and angiogenesis, eventually leading to fibrosis and wound healing. Paradoxically, these pro-inflammatory and pro-angiogenic stimuli also facilitate the survival and proliferation of residual cancer cells (4, 5). In recent years other perioperative events and conditions have been suspected of modifying the risk of metastatic disease development. Factors such as hypothermia, blood transfusion, and use of open (rather than minimally-invasive) surgical techniques are hypothesised as having detrimental effects on recurrence risk (6–8). Among these modifiable factors is the choice of anaesthetic and analgesic agents used perioperatively (9). A large volume of laboratory research has identified numerous pro- and anti-neoplastic effects associated with commonly used anaesthetic agents (10). Some retrospective clinical evidence has also pointed to a beneficial effect on cancer outcomes associated with the choice of anaesthetic used (e.g. intravenous agents such as propofol versus inhalational agents such as sevoflurane) (11, 12). There are multiple biologic pathways through which these agents may exert such effects, with modulation of the immune and inflammatory responses, as well as direct effects on cancer cells among the most likely candidates (13). The following sections will outline the perioperative use of lidocaine and our current understanding of the pathophysiology underlying postoperative cancer recurrence, before summarising recent laboratory, preclinical and clinical studies as well as planned trials examining lidocaine’s influence on cancer biology and outcomes.

## Methods

The keywords ‘lidocaine cancer’ were used to search the Medical Literature Analysis and Retrieval System (MEDLINE), Excerpta Medica database (EMBASE) and Web of Science databases. Studies from 1 January 2000 until 10 March 2021 were included as well as any referenced articles deemed significant irrespective of publication date. Randomised controlled trials, retrospective studies, meta‐analyses and systematic reviews were included. Articles were assessed for relevance and data were qualitatively analysed.

## Physicochemical Properties of Lidocaine and Clinical Uses

Lidocaine (or 2-diethylaminoaceto-2’,6’xylidide, C_14_H_22_N_2O_) is the prototype amide local anaesthetic (LA) and clinically used both as an anaesthetic and analgesic agent, as well as an anti-arrhythmic. Lidocaine principally acts by blocking voltage-gated sodium channels, preventing the rapid influx of sodium required to depolarise the cell and thereby blocking neural impulse conduction. Hence, the transmission of pain signals from peripheral tissue to the central nervous system (CNS) is blocked (14). Lidocaine also possesses activity at a wide range of other ion channels and cell receptors which potentially contributes to its observed analgesic effects (15). Compared to the other amide LAs (e.g. bupivacaine), lidocaine has a shorter half-life and is less toxic - as a result, it is the only amide LA compatible with intravenous administration. Lidocaine toxicity manifests as CNS involvement (tinnitus, altered consciousness, seizures, coma) followed by cardiac signs (arrhythmias potentially resulting in cardiac arrest). Toxicity is rare when plasma concentrations are maintained below 5 µg.ml^-1^ (~22µM) (16). In the perioperative setting, lidocaine is typically administered either systemically (intravenously) or to provide regional anaesthesia. Systemic lidocaine is given as an infusion during surgery (and often continued post-operatively) primarily for analgesic purposes; intravenous use has also been associated with faster return of gastrointestinal motility following bowel surgery, although evidence remains uncertain (17). One suggested regime consists of a maximum loading dose of 1.5 mg.kg^-1^ followed by a maximal infusion rate of 1.5 mg.kg^-1^.hr^-1^ for up to 24 hours (18), although 2 mg.kg^-1^.hr^-1^ may achieve better analgesic effects (19). Resultant plasma concentrations are in the range of 0.5 – 5 µg.ml^-1^ (2 – 22µM) (20).

## Pathophysiologic Basis of Postoperative Cancer Recurrence

### Surgery, Circulating Tumour Cells and the Pre-Metastatic Niche

**Figure 1:**
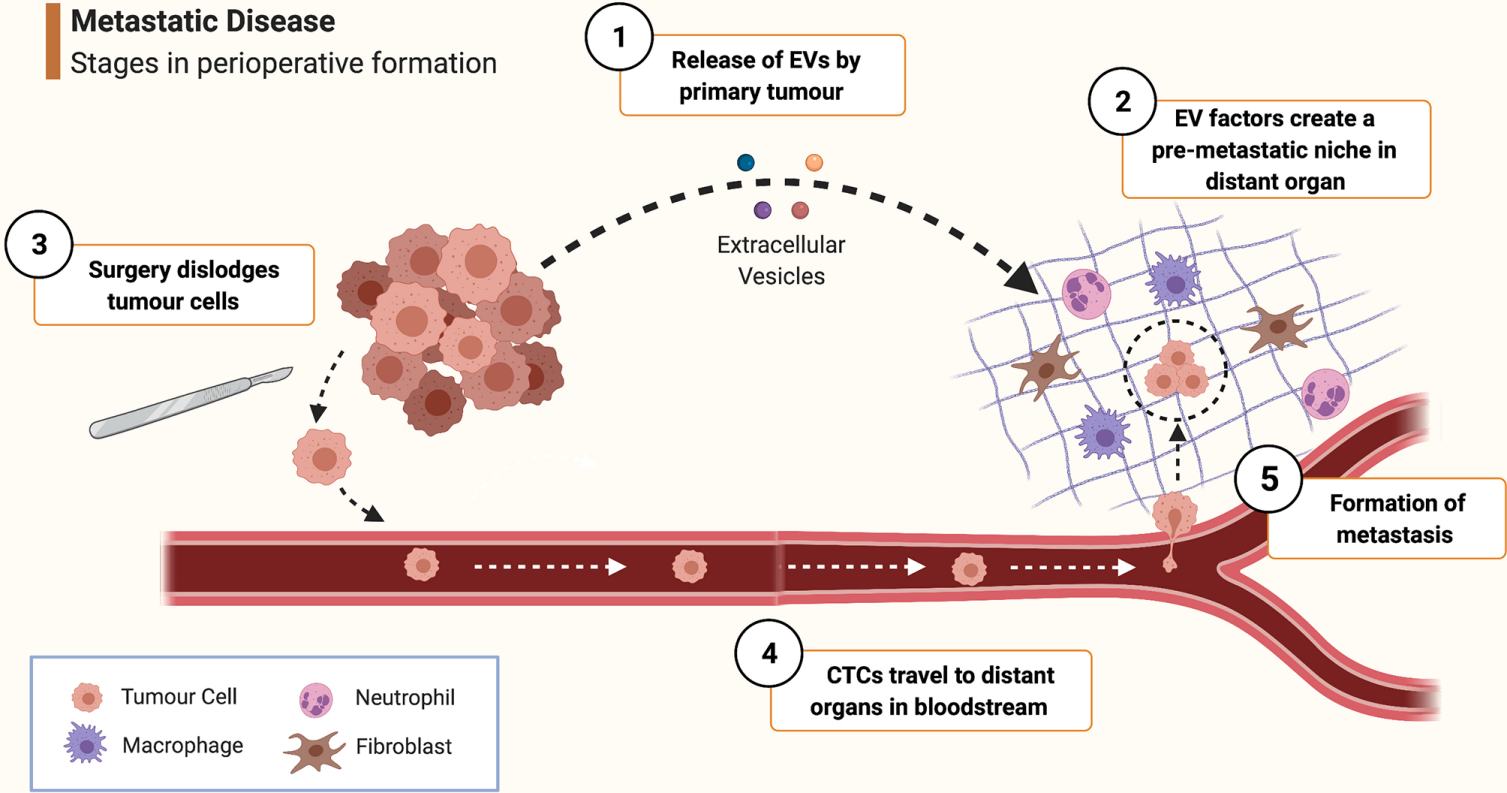
Schematic overview of pathophysiological mechanisms involved in peri-operative metastasis formation. ① As it develops, the primary tumour releases extracellular vesicles (EVs) containing growth factors, miRNAs etc. ② EV-contained factors create a pre-metastatic niche in distant organs by stimulating local cells such as fibroblasts, macrophages and mesenchymal stem cells to promote pro-neoplastic processes such as angiogenesis, inflammation and stromal remodelling. ③ During surgery, malignant cells are dispersed from the primary tumour and are released into the bloodstream to form circulating tumour cells (CTCs). ④ CTC are borne in the circulation to distant tissue beds where they arrest and extravasate into a pre-metastatic niche. ⑤ Survival conditions for the tumour cell are rendered even more favourable by the effects of mediators of the surgical stress response and inflammation, furthering the processes of angiogenesis, immune evasion etc. thus enabling the cancer cell to survive and proliferate and eventually form a clinically significant metastasis. (Created with BioRender^®^).

Metastasis begins when cancer cells are liberated from the primary tumour, enter the lymphatics or bloodstream (forming circulating tumour cells, CTCs) and subsequently seed distant tissues. Intraoperatively, CTCs may inadvertently be created when cancer cells are dislodged during tumour manipulation (**Figure 1**). Even after CTCs deposit in remote tissues, much adversity has to be overcome to successfully endure hostile immune surveillance and inadequate local homeostatic supports. Cancers, however, possess a remarkable ability to precondition distant organs to form pre-metastatic niches (PMNs) to aid the future survival and proliferation of arriving CTCs (5). PMNs are pre-programmed by extracellular vesicles (EVs) released by the primary tumour - these are cell-derived, membranous structures containing proteins, lipids, messenger RNAs and microRNAs (21, 22). MicroRNAs in particular are potent contributors to PMN formation, allowing malignancies to achieve remote ‘epigenetic regulation’ by altering gene expression in PMN cells to establish a pro-neoplastic milieu facilitating vascular permeability, angiogenesis and stromal degradation (5, 23, 24).

### Influence of Inflammation, Angiogenesis and the Surgical Stress Response on Cancer Progression

Surgery may not only disseminate tumour cells - it further promotes cancer development *via* the surgical stress response, inflammation and immunosuppression. Although vital for wound healing to occur, these physiological processes are strongly implicated in driving cancer progression; indeed, cancer has been called ‘a wound that does not heal’ (25). These processes may also cause previously formed micro-metastases to awake from dormancy and develop into significant metastatic disease. Thus excising cancerous tissue creates conditions which enhance the malignant potential of remaining cells (6).

### Inflammation

Tissue injury creates an inflammatory state necessary to recruit and activate the cellular components responsible for wound healing (10). Macrophages and dendritic cells are activated and produce chemokines and pro-inflammatory cytokines including interleukins (such as IL-1, IL-1β, IL-6, IL-8, IL-12), tumour necrosis factor alpha (TNFα), and prostaglandins (26). Rapid increases in inflammatory mediators not only promotes local tissue healing but also stimulates cancer cell survival and proliferation (27). The immune system and the sensory nervous system (SNS) are tightly integrated: pro-inflammatory cytokines modulate pain transmission, causing peripheral and central pain sensitisation, increasing SNS and hypothalamic-pituitary-adrenal (HPA) axis outflow, in turn stimulating cytokine expression by immune cells (28). Expression of numerous signalling pathway elements are altered in the post-surgical inflammatory milieu, many of which are associated with cancer progression, including enzymes such as cyclo-oxygenase-2 (COX-2) and matrix-metalloproteinases (MMPs), and transcription factors such as nuclear factor kappa-beta (NF-κB) (29). Inflammatory cytokines impair endothelial integrity and endothelial function has been demonstrated to deteriorate for several days following surgery (30). Loss of endothelial function enables leucocyte transmigration and potentially facilitates the extravasation of CTCs into remote tissues (31). The tyrosine kinase enzyme Src contributes to this process *via* its actions as an important regulator of endothelial barrier integrity (32). Src is activated by inflammatory mediators, including TNFα, resulting in disruption of tight junctions between endothelial cells and eventual loss of endothelial function (32).

### Angiogenesis

Surgical tissue injury causes localised tissue hypoxia, resulting in upregulation of hypoxia-inducible factor (HIF), in turn stimulating expression of vascular endothelial growth factor (VEGF). VEGF drives the synthesis of numerous tissue components involved in angiogenesis including integrins and extracellular matrix (33). Similarly, rapid growth of cancerous tissue creates a hypoxic cellular microenvironment, stimulating HIF and VEGF expression to create new blood vessels to supply the oxygen and nutrients necessary for further neoplastic expansion. Overexpression of HIF and VEGF is associated with poorer prognosis in certain cancer types, including pancreatic and ovarian cancer (34, 35).

### The Surgical Stress Response and Immunosuppression

The innate and adaptive components of the immune system act in unison to eliminate cancerous cells. Natural killer (NK) cells of the innate system, and T-cells (helper CD4+ Th1 cells and cytotoxic CD8+ T-cells) of the adaptive system provide cell-mediated immunity (CMI), the most important cellular anti-cancer immune response (36). This activity is influenced by post-operative pathophysiological changes - the initial inflammatory state is followed by a period of immunosuppression during which CMI is diminished (37). When the surgical stimulus activates the SNS and HPA axis, cortisol and catecholamines are released which inhibit the anti-tumour activity of NK cells and CD8+ T cells (6, 38). NK cytotoxicity is also reduced by increases in IL-6 and prostaglandin E2 (39). CMI is influenced by helper T-lymphocytes, which can be divided into two groups: Th1 cells favouring an anti-cancer CMI effect, and Th2 cells favouring antibody-mediated immunity (40). Post-operatively, Th2 proliferation increases, shifting the Th1/Th2 balance from a Th1-predominant CMI phenotype towards Th2 dominance, protecting cancer cells from immune attack (6).

Once considered relatively passive players, mounting evidence points to neutrophils having complex yet crucial roles in carcinogenesis (41). Circulating neutrophil counts are often increased by the post-operative inflammatory state, leading to an increased neutrophil-to-lymphocyte ratio (NLR) (42). NLR elevation is associated with poorer survival in some cancers – but whether this reflects causation or merely correlation is unclear (43). Circulating neutrophils can migrate into the tumour microenvironment where they adopt an anti- or pro-tumour phenotype, termed N1 and N2 respectively (44). N1 neutrophils phagocytose cancer cells whereas N2 neutrophils promote cancer in numerous ways, including reshaping stroma by expressing VEGF or MMP-9 (45). Neutrophils can also extrude decondensed chromatin to form web-like structures called neutrophil extracellular traps (NETs) (46). This process (termed NETosis) is implicated in neoplasia with elevated serum markers of NETosis associated with poorer prognosis in certain cancers, and poorer post-operative outcomes in metastatic colorectal cancer (47, 48). How NETs promote metastasis is unclear - NETs may sequester CTCs without killing them, facilitating their arrest and possibly shielding them from cytotoxic immune cells (49, 50).

## Experimental Evidence of Lidocaine’s Antineoplastic Effects

**Figure 2:**
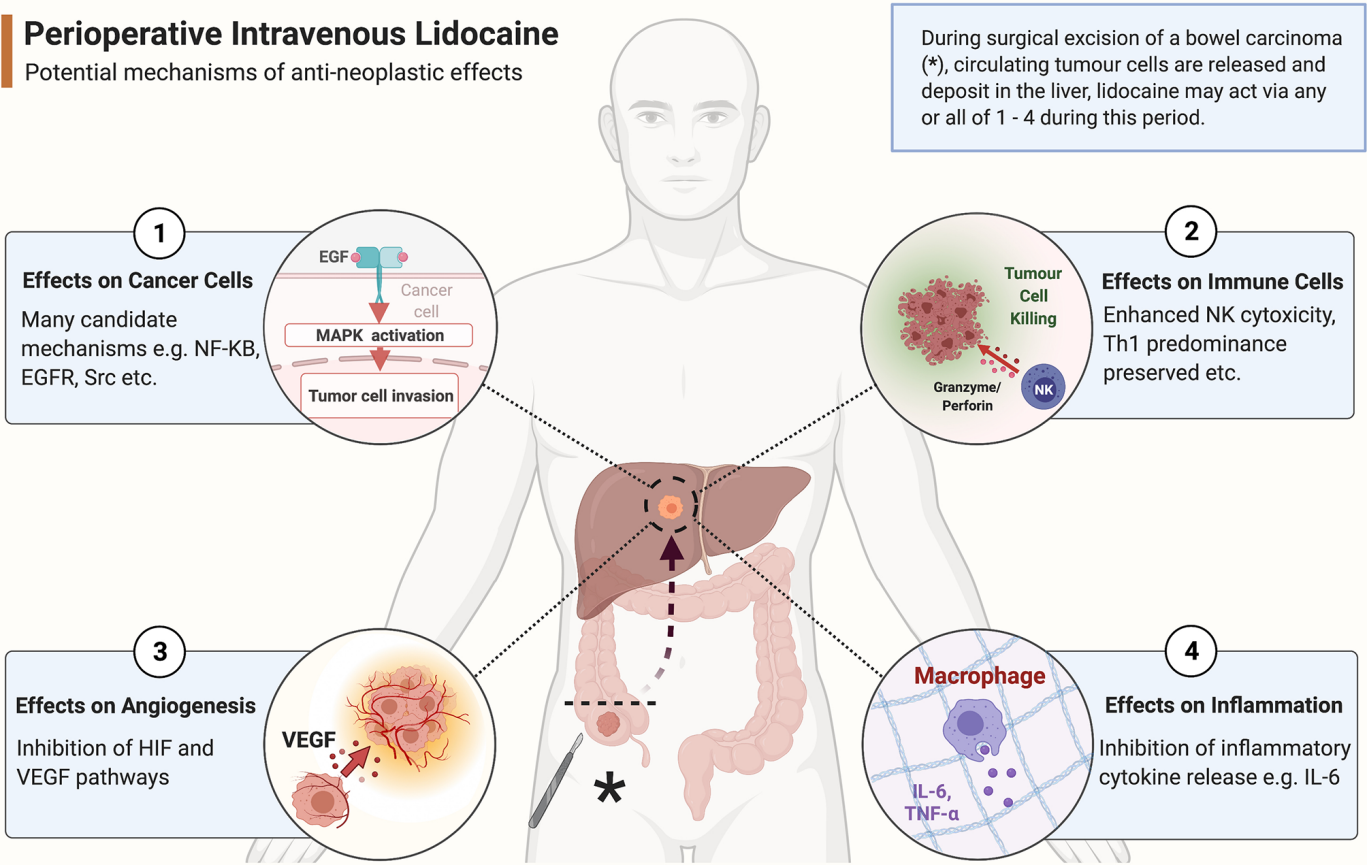
Potential anti-neoplastic mechanisms of action of systemic lidocaine during surgery. As a colonic tumour is excised (marked with *), tumour cells are released into the circulation to form circulating tumour cells (CTCs). These CTCs arrest within liver parenchyma where the likelihood of forming future clinically significant metastatic disease depends on the balance of pro- and anti-neoplastic processes present in the tumour microenvironment. Perioperative systemic lidocaine bathes the tumour cells and their microenvironment during this sensitive period and potentially beneficially alters the odds of host survival *via an* effect on any of ① - ④ outlined in the figure. (Created with BioRender^®^).

Lidocaine’s ability to inhibit cancer biology *in vitro* has been recognised for many years. Four decades ago, researchers identified that lidocaine exposure enhanced the cytotoxic effects of chemotherapeutic agents on cancer cells, with some authors attributing this phenomenon to inhibition of DNA damage repair (51). Since then, many cancers have been examined with numerous possible mechanisms of action proposed (52). To date, the accumulated evidence from many laboratory studies (**Table 1**) suggests that lidocaine possesses anti-neoplastic effects exerted *via* multiple biological pathways or components within cancer cells, and not just *via* voltage-gated sodium channels (31). In addition to direct effects on cancer cells, lidocaine also possesses anti-inflammatory properties which may modulate the pro-cancer effects of the stress response and preserve or enhance immune cell function (**Figure 2**) (82). Although *in vitro* studies are useful for establishing biological plausibility, their findings are not automatically transferrable to *in vivo* settings (83). Laboratory studies have often used human-toxic lidocaine concentrations, limiting the clinical applicability of their results. In addition, cancer in a host exists in a complex inter-relationship of cells, stroma, and cytokines, which is impossible to replicate *in vitro*. Lidocaine’s effect on cancer *in vivo* has historically been infrequently examined; however, results from several recent animal studies have supported the largely beneficial effects of lidocaine observed *in vitro* (**Table 2**).

**Table 1:** Selected *in vitro* studies examining the effects of lidocaine treatment on cancer cell biology.

First author	Year	Cancer	Anti-neoplastic lidocaine effects detected	Proposed mechanism involved
D’Agostino (53)	2018	Breast	Inhibition of cancer cell migration	Inhibited CXCL12/CXCR4 signalling
Li (54)	2018	Breast	Only high concentration (over toxic concentrations) of lidocaine inhibited affected cell viability or migration	Cancer cells arrested in S phase of cell cycle
Zhu (55)	2019	Cervical	Inhibition of cancer cell viability and promotion of apoptosis	Modulation of lncRNA-MEG3/miR-421/BTG1 signalling
Zhang (56)	2019	Chorio-carcinoma	Lidocaine stimulates apoptosis in high concentrations, potentiation of the cytotoxicity of 5-FU	Reduction of ATP-binding cassette (ABC) transport protein expression
Qu (57)	2018	CRC	Inhibition of cancer cell proliferation and promotion of apoptosis	Suppression of EGFR expression by upregulation of microRNA miR-520a-3p
Siekmann (58)	2019	CRC	High concentration (1000µM) lidocaine reduced cell proliferation but low concentrations promote cell viability in metastatic cell lines	Not assessed
Tat (59)	2019	CRC	Reduced cell proliferation	Altered expression of caspase-8, HSP-27/60, IGF-II, IGF binding protein, p53, survivin
Bundscherer (60)	2017	CRC	Cell cycle arrest induced in two CRC cell lines by 1000µM lidocaine, but no change in cell proliferation noted	Cell cycle arrest
Zhu (61)	2020	Esophageal	Decreases cell growth, migration and survival	Causes mitochondrial dysfunction and oxidative damage, anti-migratory effects linked to decreased Rac1 activity
Ye (62)	2019	Gastric	Inhibition of cancer cell proliferation, migration, invasion and promotion of apoptosis	Decreased Bcl-2 expression, increased Bax expression, alteration of MAPK pathway
Sui (63)	2019	Gastric	Reduced cell viability, proliferation, migration and invasion, promoted apoptosis	Enhanced expression of miR-145, inactivation of MEK/ERK and NF-κB pathways, downregulated Bcl-2 expression, upregulated cleaved caspase-3/-7/-9 expression, decreased MMP-2/-9
Yang (64)	2018	Gastric	Inhibition of cancer cell proliferation and migration	Down-regulation of p-ERK1/2
Zhang (65)	2020	Gastric	Lidocaine inhibited cell migration and invasion, as well as reducing resistance to cisplatin	Inhibition of B-catenin and AKT/mTOR pathways by decreased expression of miR-10b
Izdebska (66)	2019	Glioma (rat)	Increased apoptosis and necrosis of cancer cells	Cytoskeletal reorganisation, possible induction of cytoprotective autophagy
Leng (67)	2017	Glioma (rat)	Lidocaine inhibits glioma cell proliferation	Inhibition of TRPM7 currents
Liu (68)	2018	HCC	Decreased HCC cell viability and colony formation	Upregulation of cytoplasmic polyadenylation element binding protein 3 (CPEB3)
Jurj (69)	2017	HCC	Inhibition of cell proliferation	Reduced expression level of p53
Le Gac (70)	2017	HCC	Lidocaine decreased viability and proliferation of HCC cell, increased apoptosis of HCC progenitor cells	Increased mRNA of APC, an antagonist of the Wnt/B-catenin pathway
Ni (71)	2018	Leukaemia stem cells	Lidocaine inhibited proliferation and colony formation of LSCs	Inhibition of Wnt/B-catenin signalling
Sun (72)	2019	Lung	Inhibited viability, migration, invasion; promotion of apoptosis	Increased expression of miR-539, inhibited EGFR signalling
Zhang (73)	2017	Lung	Reduced cancer cell proliferation	Downregulation of GOLT1A expression
Yang (74)	2019	Lung	Lidocaine reduced cancer cell viability, migration and invasion, as well as reducing resistance of lung cancer cells to cisplatin	Down-regulation of miR-21
Piegeler (75)	2015	Lung	Lidocaine reduced cancer cell invasion	Lidocaine reduced TNFα-induced activation of Akt, FAK, caveolin-1 and reduced MMP-9 secretion.
Dong (76)	2019	Lung	Lidocaine reduced viability of lung ca cells	Increased Bax/Bad expression, decreased Bcl-2 expression
Wang (77)	2016	Lung	Lidocaine decreases viability, invasion, migration and promotes apoptosis in NSCLC cells	Downregulation of ΔΨm, provoked DNA damage, upregulated ROS production and activated MAPK pathways
Zheng (78)	2020	Melanoma	Lidocaine inhibited migration and proliferation of melanoma cells and increased apoptosis	Inhibition of small GTPases RhoA, Rac1 and Ras
Wang (79)	2017	Melanoma	Lidocaine sensitizes the cytotoxicity of 5-FU in melanoma cells	Upregulation of miR-493, potentially affecting SOX4-mediated pathways
Mirshahidi (80)	2020	Osteo-sarcoma	Lidocaine reduced viability of cancer cells, increased apoptosis	Bcl-2 and survivin expression decreased; Bax, cleaved caspase-3 and cleaved poly (ADP-ribose) polymerase-1 were increased.
Chang (81)	2014	Thyroid	Decreases cell viability and colony formation, induces apoptosis	Activation of caspase 3/7, alters ratio of Bax/Bcl-2, attenuates ERK1/2 activity, activation of MAPK

**Table 2:** Selected in *vivo* studies examining the effects of lidocaine treatment on cancer progression, metastasis or survival.

First Author	Year	Cancer	Study Type	Anti-neoplastic Lidocaine Effects Detected	Proposed Mechanism(s) Involved
Chamaraux-Tran (84)	2018	Breast	*In vitro* & in *vivo* (mouse)	Inhibition of cancer cell migration and viability; improved survival of mice with peritoneal carcinomatosis	Not assessed
Yang (85)	2018	Bladder	*in vitro* & in *vivo* (mouse)	Inhibition of cancer cell proliferation *in vitro*; *in vivo* intravesical lidocaine and mitomycin C combined prolonged survival and reduced bladder weight	Not assessed
Wall (86)	2019	Breast	*In vivo* (mouse)	Reduced post-surgical pulmonary metastasis count	Reduced MMP-2 expression
Johnson (87)	2018	Breast	*In vivo* (mouse)	Reduced post-surgical pulmonary metastasis count	Reduced pro-inflammatory and pro-angiogenic cytokine expression
Freeman (88)	2018	Breast	*In vivo* (mouse)	Decreased post-surgical pulmonary metastasis count when combined with cisplatin	No attributable change in cytokine expression detected
Freeman (89)	2018	Breast	*In vivo* (mouse)	Reduced post-surgical pulmonary metastasis count	No attributable change in cytokine expression detected
Chen (90)	2019	Melanoma	*In vitro* & in *vivo* (mouse)	Reduced cancer cell proliferation *in vitro*; in *vivo* lidocaine reduced tumour volume and weight	Cell cycle arrest in G1 phase, inhibited Ki-67 expression, inhibited ERK phosphorylation
Gao (91)	2019	Melanoma (*in vivo*)	*In vitro* (HUVEC) & in *vivo* (mouse)	In vitro lidocaine inhibited angiogenesis, *in vivo* lidocaine inhibited tumour angiogenesis and reduced tumour growth (mouse melanoma model)	Suppression of VEGF-activated phosphorylation of VEGF receptor 2 (VEGFR2), PLCγ-PKC-MAPK and FAK-paxillin
Xia (92)	2019	Retino-blastoma	*In vitro* & in *vivo* (mouse)	*In vitro* lidocaine inhibits proliferation and induces apoptosis; *in vivo* lidocaine reduces volume and weight of tumours	Increased expression of miR-520a-3p, decreased expression of EGFR
Xing (93)	2017	HCC	*In vitro* & in *vivo* (mouse)	Lidocaine inhibited HCC cell viability at higher concentrations (>0.5mM), apoptosis increased, cell arrest in G_0_/G_1_ phase; *in vitro* lidocaine inhibited tumour growth	Activation of caspase 3, decreased Bcl-2 and Bax expression, inactivation of ERK1/2 and p38 pathways

## Effects on Cancer Cell Biology

### Effects on Bax/Bak/Bcl-2 and Apoptosis

Whether a damaged or pre-cancerous cell undergoes programmed cell death or not depends on the intracellular balance between pro- and anti-apoptotic mediators. The pro-apoptotic proteins, Bax and Bak, induce the mitochondrial release of cytochrome c and other apoptosis-regulating factors (94). These in turn activate caspases (proteolytic enzymes) which degrade cellular components causing cell fragmentation and phagocytosis by macrophages (94, 95). Countering Bax and Bak is the protein Bcl-2 which exerts anti-apoptotic effects favouring cell survival (96). Aberrant regulation of these pathways is linked to carcinogenesis. Lidocaine has been shown to induce apoptosis in multiple cancer cell lines *in vitro* across numerous studies (**Table 1**). Ye et al. observed that lidocaine inhibited gastric cancer cell proliferation, migration and invasion as well as promoting apoptosis – a finding associated with decreased Bcl-2 and increased Bax expression (62). Similar lidocaine-induced alterations in the Bax/Bcl-2 ratio to favour apoptosis were also detected in lung cancer cells (76). Separate studies examining osteosarcoma, thyroid cancer and hepatocellular carcinoma cells found that lidocaine-associated apoptosis was accompanied not only by alteration of the Bax/Bcl-2 ratio but also activation of caspases (80, 81, 93).

### Effects on EGFR and the MAPK Pathway

The epidermal growth factor receptor (EGFR) is a widespread transmembrane receptor activated by a number of extracellular ligands including the mitogens EGF and TGF-α. Binding of ligands activates complex downstream signalling cascades, including mitogen-activated protein kinase (MAPK) systems such as the extracellular signal-regulated kinase (ERK1/2) and p38 pathways (97). This results in DNA transcription and promotion of processes leading to cell proliferation, migration and angiogenesis. MAPK pathways also play a role in apoptosis, where highly complex MAPK signalling may have either a pro- or anti-apoptotic effect depending on the cell type and stimulus involved (98). Defective EGFR signalling plays a major role in carcinogenesis, and many oncological therapies specifically target this signalling cascade (99). Lidocaine may also influence EGFR pathways resulting in antineoplastic effects. Researchers found lidocaine increased expression of miR-539 (an EGFR suppressor) in lung cancer cells treated *in vitro* resulting in EGFR inhibition and reduced viability, migration and invasion as well as apoptosis (72). To reinforce these findings, the anti-neoplastic effects of lidocaine were attenuated when miR-539 was silenced (72). Another miRNA-based effect on EGFR was detected when the mechanisms by which lidocaine inhibited proliferation and enhanced apoptosis in colorectal cancer cells were examined (57). In this instance miR-520a-3p directly targeted EGFR and lidocaine increased its expression (albeit at 500-1000µM). Similar lidocaine-induced alteration of the miR-520a-3p/EGFR relationship leading to anti-neoplastic effects was also noted in retinoblastoma cells (92).

Alteration of p38 and ERK1/2 pathways have been hypothesised as underlying lidocaine’s anti-cancer effects in multiple *in vitro* experiments. In one study, lidocaine was noted to induce p38 phosphorylation in gastric cancer cells alongside an increase in apoptosis and decrease in proliferation, migration and invasion - the authors hypothesising that lidocaine-activated p38-MAPK signalling was the underlying mechanism (62). Other groups detected inactivation of both p38 and ERK1/2 pathways as well as activation of caspase 3 and alteration of the Bcl-2/BAX ratio when HCC cells were treated with lidocaine; in addition, viability was reduced and apoptosis increased in exposed cells (93). Further evidence linking anti-cancer effects of lidocaine to altered ERK signalling has been found in experiments examining gastric cancer, thyroid cancer and melanoma cells (64, 81, 90).

### Effects on NF-κB

NF-κB is a protein transcription factor and regulator of numerous cellular processes occurring in response to tissue injury including immune response, inflammation, angiogenesis and apoptosis, in addition to playing a crucial role in cancer development (100). Cell stress signals (such as TNFα-receptor binding) are linked *via* intermediate steps to the translocation of the NF-κB complex into the cell nucleus whereupon transcription of potentially hundreds of target genes is activated or repressed (101). The exact nature of the resultant cellular response depends on complex, context-specific factors including cell type, cell health, and the nature of the stimulus. Adding to the complexity of NF-κB‘s functions, multiple points of crosstalk exist between the NF-κB pathway and disparate signalling pathways involving transcription factors, microRNAs and cytokines, amongst others (100).

Alteration of NF-κB signalling by lidocaine has been demonstrated in a number of cancer types. Sui et al. detected inhibitory effects of lidocaine on gastric cancer cells, a finding attributed to upregulation of miR-145 resulting in inactivation of NF-κB and MEK/ERK pathways (63). miR-145 has been hypothesised as a potential gastric cancer suppressor and indeed Sui demonstrated that transfection with an miR-145 inhibitor reversed the anti-neoplastic effects of lidocaine on the cancer cells and the NF-κB and MEK/ERK pathways (63). Lidocaine has also been shown *in vivo* (in animal models of sepsis and sterile inflammation) to inhibit expression of the inflammatory mediator high mobility group box 1 (HMGB1), which in turn suppresses activation of NF-κB (102, 103). These findings are supported by a randomised control trial (RCT) which allocated patients undergoing radical hysterectomy to intraoperative i.v. lidocaine or placebo and found that lidocaine reduced serum HMGB1 and inhibited its expression by peripheral monocytes (104). Beneficial lidocaine-related NF-κB changes have also been detected in immune cells - Lahat et al. found that lidocaine reduced nuclear NF-κB in T-cells, inhibited T-cell proliferation *in vitro* and inhibited T-cell production of the pro-inflammatory cytokines IL-2, TNFα and IFN-γ (105).

### Inhibition of the Wnt/β-catenin Pathway

Wnt pathways are complex signalling systems that direct cellular processes influencing organogenesis including cell fate determination, motility and stem cell renewal amongst others (106). β-catenin is a crucial component of the ‘canonical’ or Wnt/β-catenin pathway and acts as a transducer of this signalling mechanism which determines cell proliferation. Dysregulation of Wnt pathways is associated with development of numerous malignancies including colorectal cancer (107). The protein known as adenomatous polyposis coli (APC) contributes to the formation of the β-catenin destruction complex which degrades β-catenin leading to reduced Wnt/β-catenin signalling thereby inhibiting cell proliferation and migration (108). Recent experimental evidence has suggested that lidocaine’s observed *in vitro* antineoplastic properties are potentially related to an effect on the Wnt/β-catenin pathway. One study identified that lidocaine increases the mRNA of the β-catenin antagonist APC ten-fold when applied to HCC cells, a finding associated with decreased cell viability and proliferation (70). Lidocaine repressed Wnt/β-catenin activity in two other *in vitro* studies examining gastric cancer and leukaemia cells respectively (65, 71). To the best of our knowledge, lidocaine’s effect on this pathway has yet to be examined in an animal model.

### Inhibition of Transient Receptor Potential Channels

Transient receptor potential (TRP) channels are a large family of widely expressed membrane ion channels, playing a role in cell growth, survival and proliferation (109). Several TRP family members (including TRPV1, TRPV6 and TRPM7) have been associated with oncogenesis, and increased TRP expression correlates negatively with tumour grade and patient survival in some cancers (110). Lidocaine can inhibit TRPM7 channel current, and TRMP7 suppression is associated with reduced proliferation, migration and invasion of glioma and breast cancer cells *in vitro* (67, 111–113). Similarly, lidocaine reduced TRPV6 expression, migration and invasion in TRPV6-positive breast, prostate and ovarian cancer cells (114). Lidocaine also increased apoptosis in glioma cells, an effect attributed to activation of the TRPV1 gene (115).

### Effects on Src Signalling

Src is a non-receptor tyrosine kinase protein widespread in human cells and its encoding gene was the first proto-oncogene to be identified (116). Src is activated by various stimuli, such as TNFα binding to its receptor; activated Src phosphorylates a range of targets including the membrane protein caveolin-1. Src activation results in reduced endothelial barrier function and promotes cellular survival, proliferation, migration, invasion and angiogenesis (32, 117). Predictably then, activated Src in tumour cells is a potent oncogenic promoter and drives the pathogenesis of multiple cancers including colon, prostate and breast carcinomas (118, 119). Activated Src induces the expression of the enzymes MMP-2 and -9 which degrade basement membrane, thereby facilitating tumour cell migration and invasion (120). The effects of lidocaine on Src and associated signalling by-products have been studied both *in vitro* and *in vivo*. In separate experiments Piegeler et al. examined lung adenocarcinoma and lung endothelial cells *in vitro* and demonstrated that lidocaine not only inhibited TNFα-induced Src activation in both cell types, but also reduced cancer cell migration and endothelial cell permeability, as well as neutrophil adhesion (121, 122). In a subsequent study, the same group showed that lidocaine-related inhibition of TNFα-induced, Src-dependent signalling in lung adenocarcinoma cells resulted in reduced MMP-9 expression and reduced cancer cell invasion (75). Although Src inhibition by lidocaine has consistently demonstrated anti-tumour effects *in vitro*, this effect is yet to be confirmed *in vivo*. Our group examined whether an effect on Src underpinned lidocaine-related inhibition of pulmonary metastasis in a mouse model of breast cancer surgery by introducing an Src inhibitor alongside lidocaine. Although postoperative serum MMP-2 was reduced in lidocaine-treated animals, the results could not confirm an Src-dependent mechanism (86).

## Effects on Inflammatory Cytokine Production

Lidocaine has long been known to possess anti-inflammatory properties (82). What has been more difficult to determine is the mechanism(s) by which inflammation is suppressed and the resulting clinical significance, if any. In vitro evidence from a number of studies has demonstrated that lidocaine inhibits release of leukotrienes, histamine and IL-1α from leukocytes - all potent inflammatory mediators (123, 124). Lidocaine may also inhibit the ‘priming’ or potentiation of neutrophil response to certain triggers of inflammation and thus reduce cytokine expression (125). In addition, lidocaine experimentally inhibits immune cell adhesion, migration and proliferation within areas of tissue injury (126). This may result from a protective effect of lidocaine on endothelium, preventing inflammatory mediator-induced injury and thus preserving endothelial barrier integrity (127). Conceptually then, perioperative lidocaine may inhibit immune cell infiltration into the pre-metastatic niche and prevent such cells releasing pro-metastatic inflammatory cytokines into this nascent tumour microenvironment, so reducing the risk of future metastasis development.

A number of small RCTs have examined the effect of i.v. lidocaine on post-operative cytokine expression (**Table 3**). Ortiz et al. randomised laparoscopic cholecystectomy patients (n=44) to receive either i.v. lidocaine (1.5 mg.kg^-1^ bolus at surgical start then 3 mg.kg^-1^.h^-1^ until 1 hour after surgery) or i.v. saline as placebo (128). At 24 hours post-surgery compared to those receiving saline, i.v. lidocaine recipients had significantly reduced serum levels of pro-inflammatory cytokines (IL-1, IL-6, TNFα, IFN-γ) and an increase in the anti-inflammatory cytokine IL-10 suggestive of an overall anti-inflammatory effect. 5 other RCTs have detected that lidocaine has an inhibitory effect on serum cytokine concentrations following abdominal surgery, with IL-6 expression the most consistently suppressed; effects on clinical cancer outcomes were not assessed (129–133).

**Table 3:** Selected RCTs comparing the effects of systemic lidocaine versus saline placebo on serum cytokine concentration.

First Author	Year	Surgery Type & No. Recruited	I.V. Lidocaine Dosing	Effects on Postoperative Serum Inflammatory Marker Concentrations
Ortiz (128)	2016	Laparoscopic cholecystectomy (n=44, 22 per group)	1.5 mg.kg^-1^ bolus then 3 mg.kg^-1^.h^-1^ until 1 hour post-surgery	IL-1, IL-6, IFN-γ, and TNFα reduced in i.v. lidocaine group and IL-10 increased compared with placebo group
Song (129)	2017	Laparoscopic cholecystectomy (n=80, 40 per group)	1.5 mg.kg^-1^ bolus then 2 mg.kg^-1^.h^-1^ until end of surgery	IL-6 and IL-8 reduced in i.v. lidocaine group and no effect on IL-1ra compared with saline placebo
Kuo (130)	2006	Colon cancer surgery (n=60, 30 per group)	2 mg.kg^−1^ bolus then 3 mg.kg^−1^.h^−1^ until end of surgery	IL-6, IL-8 and IL-1ra reduced by both i.v. and epidural lidocaine compared with saline placebo
Herroeder (131)	2007	Colorectal surgery (n=60, 30 per group)	1.5 mg.kg^-1^ bolus then 2 mg.kg^-1^.h^-1^ until 4 hours post-surgery	Lidocaine attenuated increase of IL-6 and IL-8, no effect on IL-1β and TNF-α
Yardeni (132)	2009	Open hysterectomy (n=65, 32/33 in each group)	2 mg.kg^−1^ bolus then 1.5 mg.kg^−1^.h^−1^ until end of surgery	Lidocaine attenuated the increase of IL-6 and IL-1ra produced by lipopolysaccharide–stimulated peripheral blood mononuclear cells
Sridhar (133)	2015	Open abdominal surgery (n=134, 67 per group)	2 mg.kg^−1^ bolus then 1.5 mg.kg^−1^.h^−1^ until 1 hour post-surgery	Lidocaine attenuated IL-6 compared to saline placebo
Dewinter (134)	2017	Spinal surgery (n=70, 35 per group)	1.5 mg.kg^-1^ bolus then 1.5 mg.kg^-1^.h^-1^ until 6 hours post-surgery	No significant differences between IL-6 and IL-1ra between the lidocaine and placebo groups
van den Heuvel (135)	2020	Breast cancer surgery (n=48, 24 received lidocaine)	1.5 mg.kg^-1^ bolus then 2 mg.kg^-1^.h^-1^ until 1 hour post-surgery	No effect attributed to lidocaine on serum IL-1β, IL-6, IL-10, IL-1ra
de Oliveira (136)	2015	Open hysterectomy (n=40, 20 per group)	No bolus, 2 mg.kg^-1^.h^-1^ infusion during surgery	No difference in serum IL-6 detected
Xu (137)	2021	Laparoscopic hysterectomy (n=160, 4 groups of 40)	1.5 mg.kg^-1^ bolus then 1.5 mg.kg^-1^.h^-1^ until 30 mins before end of surgery	No difference in serum IL-1, IL-6 and TNF-α between control group receiving saline and group receiving lidocaine

Not every RCT published to date has demonstrated lidocaine-related anti-inflammatory effects. Similar studies examining breast surgery, spinal surgery and hysterectomy patients found no difference in post-operative serum cytokines in their lidocaine treatment arms (134, 135, 137). There may be a number of reasons underlying the variable results observed in these trials. The enrolled numbers in the RCTs performed were small and most were powered to detect clinical outcomes (such as pain) as the primary outcome rather than cytokine changes. Significant heterogeneity existed not only in the dose and duration of infusion administered, but also in the time points at which cytokines were measured. Notably, all the RCTs reporting lidocaine-related cytokine reductions examined abdominal surgery, and indeed lidocaine’s clinical benefits in terms of analgesic effects, hastening return of bowel function and decreasing hospital stay appear most pronounced in this cohort (16).

## Effects on Angiogenesis

Given that inflammation and angiogenesis are often inextricably linked, it is difficult experimentally to isolate purely angiogenic pathways from inflammatory ones (138). There is significant overlap between the intracellular signalling pathways activated by both hypoxia and inflammation – hypoxia inducible factors (HIFs) increase transcription of NF-κB in the same way that inflammatory stimuli do, leading to amplification of inflammatory mediator production, as well as increasing expression of pro-angiogenic VEGF (138). The effects of lidocaine on HIF or VEGF specifically has infrequently been studied in laboratory or preclinical experiments. Gao et al. examined endothelial cells *in vitro* and found that VEGF-stimulated cell migration and proliferation was inhibited by lidocaine (50µM), as well as suppression of VEGF/VEGF receptor 2 (VEGFR2) signalling at 100µM (91). Using a mouse melanoma model, the same group found that intraperitoneal lidocaine treatment resulted in smaller tumours with reduced blood vessel formation. Separately, Suzuki et al. detected similar lidocaine-associated anti-angiogenic effects on endothelial cells *in vitro* although at lower concentrations (4 - 44µM), with similar suppression of VEGF/VEGFR2 signalling noted (139). In contrast, Nishi et al. reported that lidocaine (lowest concentration 30µM) did not affect hypoxia-induced HIF activation or alter expression of hypoxia-induced genes (140). Although choice of anaesthetic technique can alter post-operative serum VEGF in certain cohorts of cancer patients, the clinical significance of any such change is unknown and no definite effect on cancer outcomes has been proven (141, 142). Only one RCT has examined the effect of systemic lidocaine on serum VEGF (though not as the primary outcome): breast cancer surgery patients (n=120) were randomised to anaesthesia using propofol or sevoflurane, with or without i.v. lidocaine – post-operative serum VEGF concentrations were unaffected by any treatment (143).

## Effects on Immune Cells

The ability of some anaesthetic and analgesic agents to modify immune cell numbers and function is supported by laboratory evidence, although definitive clinical evidence of effects on patient outcomes is not confirmed (13, 38). Similarly, experimental evidence has accumulated indicating that lidocaine may modulate various cellular components of the immune system (31). As immune function and inflammation are closely associated, this effect may result from lidocaine’s anti-inflammatory properties as outlined previously. Or it may result from a direct action of lidocaine on immune cells, or indirectly *via* effects on SNS or HPA axis activity, or from some combination of these. Systemic lidocaine reduced circulating cortisol levels in parturients undergoing caesarean section in one trial, and post-operative urinary catecholamines in cholecystectomy patients in another (144, 145). Conversely, this effect was not observed in studies examining cortisol and/or catecholamine levels in colectomy or hysterectomy patients (146, 147). Based on this admittedly small body of evidence, SNS/HPA suppression cannot convincingly be identified as the primary means by which lidocaine influences immune cells.

Dendritic cells and macrophages treated with lidocaine *in vitro* express reduced amounts of inflammatory cytokines, a potentially beneficial anti-inflammatory and anti-cancer effect (148, 149). Conversely, lidocaine-related suppression of Th1 differentiation was detected both *in vitro* and in a mouse model, a potentially detrimental effect as Th1 cells contribute to cell mediated immunity (CMI). Clinically achievable concentrations of lidocaine may also benefit CMI by enhancing the cytotoxic effects of NK cells - *in vitro* NK cytotoxicity against leukaemia cells was promoted by lidocaine treatment (at 0.01µM to 50µM), an effect attributed experimentally to enhanced lytic granule release (150). Similar NK cytotoxicity enhancement was identified in a study which isolated NK cells from healthy donors and cancer patients (both pre- and post-operatively) - NK cells treated *in vitro* with lidocaine had greater cytotoxic effects against cancer cells (151).

A small RCT randomised 30 patients undergoing radical hysterectomy to i.v. lidocaine (1.5 mg.kg^-1^ bolus then 1.5 mg.kg^-1^.h^-1^ during surgery) or saline (152). Lidocaine treatment preserved post-operative lymphocyte proliferation and inhibited apoptosis. Another RCT, again involving hysterectomy patients (n=65), randomised subjects to i.v. lidocaine (2 mg.kg^-1^ bolus then 2 mg.kg^-1^.h^-1^ during surgery) or saline (132). Again an immune protective effect was detected - lidocaine attenuated suppression of the lymphocyte proliferative response, in addition to inhibiting expression of IL-6 and IL-1ra. Not all clinical studies have demonstrated beneficial immune effects - i.v. lidocaine (1 mg.kg^-1^ bolus) in patients with herpes zoster-related pain did not affect NK numbers or activity (153).

Lidocaine inhibits neutrophil adhesion and migration *in vitro*, with effects on the integrin member CD11b-CD18 or the Nav1.3 voltage-gated sodium channel among the mechanisms postulated (154, 155). Evidence of lidocaine’s effects on neutrophils has also been established by several animal and human studies. One *in vivo* study found that lidocaine (administered intra-peritoneally in a mouse peritonitis model) inhibited neutrophil apoptosis and macrophage clearance and delayed the resolution of the inflammatory response and return to normal homeostasis (156). Systemic lidocaine also inhibited leukocyte accumulation in animal models of peritonitis and myocardial ischaemia (157, 158). Clinical evidence is limited – in an RCT conducted by Berger et al., intravenous lidocaine administered to septic patients reduced chemokine-induced adhesion and transmigration of neutrophils through endothelium without affecting expression of adhesion molecules (159).

Lidocaine has long been recognised as affecting neutrophil phagocytic function (160), although accumulated evidence appears contradictory as to whether function is enhanced or impaired. Kawasaki et al. treated human neutrophils with lidocaine (at supraclinical 400µM) *in vitro* and found respiratory burst and phagocytic ability were impaired (161). Similar effects on respiratory burst have also been reported in neutrophils isolated from umbilical cord blood from newborns and treated *in vitro* with high concentration lidocaine (4mM), whereas low concentrations (2µM) appeared to increase reactive oxygen species production (162). However, other groups did not detect any lidocaine-related effect on *in vitro* neutrophil function or reactive oxygen species production when clinically achievable concentrations were tested (163–165). One clinical study examining neutrophils taken from lidocaine-treated patients detected significantly reduced superoxide anion release compared to patients who didn’t receive lidocaine (166). Contrary to this finding, a clinical trial studying bolus lidocaine (1.5 mg.kg^-1^) administered at induction of anaesthesia found that lidocaine actually preserved neutrophil respiratory burst compared to neutrophils from control patients who received saline (167).

The choice of anaesthetic technique can modulate the neutrophil-to-lymphocyte ratio (NLR) post-operatively, however significant effects on clinical outcomes are not proven (168, 169). Evidence from one small RCT also suggests beneficial effects of lidocaine on post-operative NLR following breast cancer surgery although, again, clinical outcomes were not assessed (170). Unsurprisingly given the relatively recent discovery of the phenomenon of NETosis, it has infrequently been studied in the context of cancer surgery. However, one RCT found that i.v. lidocaine reduced serum biomarkers of NETosis (namely neutrophil myeloperoxidase and citrullinated histone H3) after breast cancer surgery (143).

## Preclinical Studies of Cancer Outcomes

Although to date far fewer preclinical studies have been conducted than laboratory experiments, a number of animal studies have identified beneficial effects of lidocaine on *in vivo* cancer growth and outcomes (**Table 2**). Chamaraux-Tran et al. injected immunodeficient mice intraperitoneally with human breast cancer cells, randomised the animals to weekly intra-peritoneal lidocaine or saline treatment, and demonstrated that lidocaine treatment significantly improved survival and reduced tumour growth (84). Similarly, lidocaine treatment has been proven to reduce tumour size and improve survival when administered intravesically alongside mitomycin C in a mouse model of bladder cancer (85). Lidocaine also decreased tumour size when administered intraperitoneally in mouse models of melanoma and hepatocellular carcinoma, and intravenously in models of melanoma and retinoblastoma (90–93). We previously established a syngeneic mouse breast cancer model to mimic the effects of anaesthesia and surgery on postoperative metastatic progression (87). In this model, animals that received an intravenous lidocaine infusion alongside sevoflurane anaesthesia during resection of primary breast tumours had consistently fewer pulmonary metastases when measured two weeks postoperatively (86, 88, 89).

## Clinical Studies of Cancer Outcomes

Following reports from retrospective analyses suggesting decreased cancer recurrence rates associated with regional anaesthetic techniques in breast and prostate cancer surgery, there has been an increased focus on establishing which anaesthetic technique, if any, provides the greatest outcome benefit following surgery (171, 172). Evidence accumulated from laboratory and retrospective clinical studies suggests that intravenous (i.e. propofol-based) and regional anaesthesia are potentially beneficial in terms of effects on cancer outcomes compared to volatile anaesthesia and opioids (10). However, the first large RCT examining this topic, which randomised breast cancer surgery patients to a propofol-regional anaesthesia technique versus a volatile-opioid technique, found no difference in recurrence rates between the two groups (173). Given the huge degree of biological heterogeneity between different malignancies, it is difficult to determine how applicable these findings may be to other cancer surgery types e.g. colorectal cancer surgery. Other trials currently underway, assessing anaesthetic technique and cancer outcomes across a range of different cancer types, will go some way towards addressing this uncertainty.

Although numerous studies have examined the effects of intravenous lidocaine on biochemical or haematological markers of inflammation, angiogenesis and immune function, to the best of our knowledge, only one study to date has reported on clinical outcomes. Zhang et al. in a recent retrospective study of 2239 patients undergoing resection of pancreatic carcinomas found that those who received perioperative i.v. lidocaine (1.5 mg.kg^-1^ bolus followed by 2 mg.kg^-1^.hr^-1^) had significantly better overall survival at 1 and 3 years (68.0% *vs* 62.6%, p<0.001; 34.1% *vs* 27.2%, p=0.011), although disease-free survival was unaffected (174).

## Future RCTs - Establishing Systemic Lidocaine’s Effect on Cancer Outcomes

The question of whether perioperative systemic lidocaine has any influence on postoperative cancer outcomes can only be answered by the completion of a suitably powered RCT. No such trial has ever been completed, which is understandable considering the cost, patient number and length of follow up required. However, this question will be addressed for a subset of cancers by the Volatile Anaesthesia and Perioperative Outcomes Related to Cancer trial (VAPOR-C, NCT04316013) which is planned to complete in 2025. VAPOR-C will recruit 5736 colorectal and lung cancer patients and in a 2x2 factorial study randomise them to either sevoflurane or propofol anaesthesia, plus lidocaine infusion (1.5 mg.kg^-1^ bolus followed by 2 mg.kg^-1^.hr^-1^ for 4 hours then 1.5 mg.kg^-1^.hr^-1^ thereafter) or saline placebo (175). The primary outcome measure will be disease-free survival, with overall survival as a secondary endpoint.

The ALLEGRO RCT (ISRCTN52352431), which is currently ongoing and aims to recruit 562 patients, is examining the effect of systemic lidocaine (1.5 mg.kg^-1^ bolus followed by 1.5 mg.kg^-1^.hr^-1^ for 6 or 12 hours) during colorectal surgery on post-operative bowel function. Cancer outcomes will be also be studied up to 10 years post enrolment, although these are tertiary endpoints so will likely be underpowered but will potentially be a useful addition to the knowledge base (176). Other small trials are examining perioperative systemic lidocaine and cancer outcomes in colorectal surgery (NCT02786329) and pancreatic surgery (NCT04449289).

## Licencing and Safety Concerns

The appropriateness of intravenous use of lidocaine given the potential risks and as yet inconclusive benefits has recently been questioned (177). Lidocaine remains unlicensed for intravenous use for analgesic purposes, although many drugs used routinely in anaesthesia are similarly used in an ‘off-label’ manner. The likelihood of encountering toxicity appears very small when carefully dosed and under continuous monitoring, with one surgical unit reporting over 2200 patients treated with perioperative i.v. lidocaine with no reported adverse effects (20). Despite this, the potential for toxicity can never be completely excluded and therefore the potential risks and benefits of systemic lidocaine should be carefully considered by the practitioner for each patient prior to commencing treatment. Recently published dosage guidelines may aid in ensuring safe practice, with dosages reduced accordingly (or usage avoided) in the presence of conditions known to enhance toxicity (18). In addition, as recently proposed, adoption of institutional guidelines regarding administration, monitoring, detection and treatment of systemic toxicity appears prudent wherever i.v. lidocaine is administered, and training of all involved staff should be mandatory (178). Perhaps, as recently suggested by Pandit and McGuire, use of intravenous lidocaine is currently best confined to subjects participating in clinical trials (including VAPOR-C) under rigorous safety conditions and where the results of usage can contribute to establishing definitive evidence of clinical benefits or otherwise (179).

## Conclusion

The cancer patient’s perioperative course is increasingly recognised as a period during which future malignant progression may be influenced for better or worse. Cancer progression appears dependent on the development of a harmful imbalance between pro- and anti-neoplastic humoral and cellular effects, in favour of the malignancy. Circulating tumour cells released by dissection, which under normal conditions would be eradicated by the immune surveillance system, may instead establish themselves in pre-metastatic niches in distant organs, where their survival is facilitated by the pathophysiological effects generated by the surgical insult. Or pre-established micro-metastatic deposits may be woken from their dormancy in the tumour microenvironment by the same processes. Any intervention made during this critical time which can rebalance these systems in favour of host survival holds tremendous promise for improving patient outcomes. Lidocaine has been shown experimentally to possess numerous beneficial effects, potentially affecting multiple biological pathways to act as an anti-inflammatory, immune cell modulator and/or direct inhibitor of cancer cells. An intravenous infusion of lidocaine administered perioperatively may act as a simple, inexpensive and effective chemotherapeutic agent in addition to its potential analgesic properties. Only evidence from adequately powered, randomised, controlled clinical trials will confirm lidocaine’s efficacy in improving cancer outcomes - the planned VAPOR-C trial should go some way towards establishing this.

## Author Contributions

Conceptualization: TW and DB. Writing – Original Draft Preparation: TW and DB. Writing – Review & Editing: TW and DB. All authors contributed to the article and approved the submitted version.

## Conflict of Interest

The authors declare that the research was conducted in the absence of any commercial or financial relationships that could be construed as a potential conflict of interest.

## Publisher’s Note

All claims expressed in this article are solely those of the authors and do not necessarily represent those of their affiliated organizations, or those of the publisher, the editors and the reviewers. Any product that may be evaluated in this article, or claim that may be made by its manufacturer, is not guaranteed or endorsed by the publisher.

## References

[1] Bray F Ferlay J Soerjomataram I Siegel RL Torre LA Jemal A. Global Cancer Statistics 2018: GLOBOCAN Estimates of Incidence and Mortality Worldwide for 36 Cancers in 185 Countries. CA Cancer J Clin (2018) 68(6):394–424. 10.3322/caac.21492 30207593

[2] Sullivan R Alatise OI Anderson BO Audisio R Autier P Aggarwal A. Global Cancer Surgery: Delivering Safe, Affordable, and Timely Cancer Surgery. Lancet Oncol (2015) 16(11):1193–224. 10.1016/S1470-2045(15)00223-5 26427363

[3] Mehlen P Puisieux A. Metastasis: A Question of Life or Death. Nat Rev Cancer (2006) 6(6):449–58. 10.1038/nrc1886 16723991

[4] Alieva M van Rheenen J Broekman MLD. Potential Impact of Invasive Surgical Procedures on Primary Tumor Growth and Metastasis. Clin Exp Metastasis (2018) 35(4):319–31. 10.1007/s10585-018-9896-8 PMC6063335 29728948

[5] Peinado H Zhang H Matei IR Costa-Silva B Hoshino A Rodrigues G. Pre-Metastatic Niches: Organ-Specific Homes for Metastases. Nat Rev Cancer (2017) 17(5):302–17. 10.1038/nrc.2017.6 28303905

[6] Hiller JG Perry NJ Poulogiannis G Riedel B Sloan EK. Perioperative Events Influence Cancer Recurrence Risk After Surgery. Nat Rev Clin Oncol (2018) 15(4):205–18. 10.1038/nrclinonc.2017.194 29283170

[7] Horowitz M Neeman E Sharon E Ben-Eliyahu S. Exploiting the Critical Perioperative Period to Improve Long-Term Cancer Outcomes. Nat Rev Clin Oncol (2015) 12(4):213–26. 10.1038/nrclinonc.2014.224 PMC5497123 25601442

[8] Cata JP Lasala J Pratt G Feng L Shah JB. Association Between Perioperative Blood Transfusions and Clinical Outcomes in Patients Undergoing Bladder Cancer Surgery: A Systematic Review and Meta-Analysis Study. J Blood Transfus (2016) 2016:9876394. 10.1155/2016/9876394 26942040 PMC4752988

[9] Byrne K Levins KJ Buggy DJ. Can Anesthetic-Analgesic Technique During Primary Cancer Surgery Affect Recurrence or Metastasis? Can J Anaesth (2016) 63(2):184–92. 10.1007/s12630-015-0523-8 26497721

[10] Wall T Sherwin A Ma D Buggy DJ. Influence of Perioperative Anaesthetic and Analgesic Interventions on Oncological Outcomes: A Narrative Review. Br J Anaesth (2019) 123(2):135–50. 10.1016/j.bja.2019.04.062 PMC6676329 31255291

[11] Wigmore TJ Mohammed K Jhanji S. Long-Term Survival for Patients Undergoing Volatile Versus IV Anesthesia for Cancer Surgery: A Retrospective Analysis. Anesthesiology (2016) 124(1):69–79. 10.1097/ALN.0000000000000936 26556730

[12] Yap A Lopez-Olivo MA Dubowitz J Hiller J Riedel B. Anesthetic Technique and Cancer Outcomes: A Meta-Analysis of Total Intravenous Versus Volatile Anesthesia. Can J Anaesth (2019) 66(5):546–61. 10.1007/s12630-019-01330-x 30834506

[13] Duff S Connolly C Buggy DJ. Adrenergic, Inflammatory, and Immune Function in the Setting of Oncological Surgery: Their Effects on Cancer Progression and the Role of the Anesthetic Technique in Their Modulation. Int Anesthesiol Clin (2016) 54(4):48–57. 10.1097/AIA.0000000000000120 27648890

[14] Weinberg L Peake B Tan C Nikfarjam M. Pharmacokinetics and Pharmacodynamics of Lignocaine: A Review. World J Anesthesiol (2015) 4:17–29. 10.5313/wja.v4.i2.17

[15] Hermanns H Hollmann MW Stevens MF Lirk P Brandenburger T Piegeler T. Molecular Mechanisms of Action of Systemic Lidocaine in Acute and Chronic Pain: A Narrative Review. Br J Anaesth (2019) 123(3):335–49. 10.1016/j.bja.2019.06.014 31303268

[16] McCarthy GC Megalla SA Habib AS. Impact of Intravenous Lidocaine Infusion on Postoperative Analgesia and Recovery From Surgery: A Systematic Review of Randomized Controlled Trials. Drugs (2010) 70(9):1149–63. 10.2165/10898560-000000000-00000 20518581

[17] Weibel S Jelting Y Pace NL Helf A Eberhart LH Hahnenkamp K. Continuous Intravenous Perioperative Lidocaine Infusion for Postoperative Pain and Recovery in Adults. Cochrane Database Syst Rev (2018) 6:Cd009642. 10.1002/14651858.CD009642.pub3 29864216 PMC6513586

[18] Foo I Macfarlane AJR Srivastava D Bhaskar A Barker H Knaggs R. The Use of Intravenous Lidocaine for Postoperative Pain and Recovery: International Consensus Statement on Efficacy and Safety. Anaesthesia (2021) 76(2):238–50. 10.1111/anae.15270 33141959

[19] Moyano J Giraldo SP Thola LM. Use of Intravenous Lidocaine for Postoperative Pain and Recovery. Anaesthesia (2021) 76(5):721. 10.1111/anae.15434 33591570

[20] Greenwood E Nimmo S Paterson H Homer N Foo I. Intravenous Lidocaine Infusion as a Component of Multimodal Analgesia for Colorectal Surgery-Measurement of Plasma Levels. Perioper Med (Lond) (2019) 8:1. 10.1186/s13741-019-0112-4 30858969 PMC6390549

[21] Braicu C Tomuleasa C Monroig P Cucuianu A Berindan-Neagoe I Calin GA. Exosomes as Divine Messengers: Are They the Hermes of Modern Molecular Oncology? Cell Death Differ (2015) 22(1):34–45. 10.1038/cdd.2014.130 25236394 PMC4262777

[22] Fares J Fares MY Khachfe HH Salhab HA Fares Y. Molecular Principles of Metastasis: A Hallmark of Cancer Revisited. Signal Transduction Targeted Ther (2020) 5(1):28. 10.1038/s41392-020-0134-x PMC7067809 32296047

[23] Tao SC Guo SC. Role of Extracellular Vesicles in Tumour Microenvironment. Cell Commun Signal (2020) 18:163. 10.1186/s12964-020-00643-5 33081785 PMC7574205

[24] Fabian MR Sonenberg N Filipowicz W. Regulation of mRNA Translation and Stability by microRNAs. Annu Rev Biochem (2010) 79:351–79. 10.1146/annurev-biochem-060308-103103 20533884

[25] Dvorak HF. Tumors: Wounds That do Not Heal. Similarities Between Tumor Stroma Generation and Wound Healing. N Engl J Med (1986) 315(26):1650–9. 10.1056/NEJM198612253152606 3537791

[26] Relja B Land WG. Damage-Associated Molecular Patterns in Trauma. Eur J Trauma Emergency Surg (2020) 46(4):751–75. 10.1007/s00068-019-01235-w PMC7427761 31612270

[27] Szalayova G Ogrodnik A Spencer B Wade J Bunn J Ambaye A. Human Breast Cancer Biopsies Induce Eosinophil Recruitment and Enhance Adjacent Cancer Cell Proliferation. Breast Cancer Res Treat (2016) 157(3):461–74. 10.1007/s10549-016-3839-3 PMC5026505 27249999

[28] Miller RJ Jung H Bhangoo SK White FA. Cytokine and Chemokine Regulation of Sensory Neuron Function. Handb Exp Pharmacol (2009) 194):417–49. 10.1007/978-3-540-79090-7_12 PMC2746245 19655114

[29] Sethi G Shanmugam MK Ramachandran L Kumar AP Tergaonkar V. Multifaceted Link Between Cancer and Inflammation. Biosci Rep (2012) 32(1):1–15. 10.1042/BSR20100136 21981137

[30] Hu YJ Wei AN Chook P Yin Y Cheng W Wu MJ. Impact of non-Cardiovascular Surgery on Reactive Hyperaemia and Arterial Endothelial Function. Clin Exp Pharmacol Physiol (2013) 40(7):466–72. 10.1111/1440-1681.12111 23662794

[31] Chamaraux-Tran TN Piegeler T. The Amide Local Anesthetic Lidocaine in Cancer Surgery-Potential Antimetastatic Effects and Preservation of Immune Cell Function? A Narrative Review. Front Med (Lausanne) (2017) 4:235. 10.3389/fmed.2017.00235 29326939 PMC5742360

[32] Hu G Minshall RD. Regulation of Transendothelial Permeability by Src Kinase. Microvasc Res (2009) 77(1):21–5. 10.1016/j.mvr.2008.10.002 19027754

[33] Darby IA Hewitson TD. Hypoxia in Tissue Repair and Fibrosis. Cell Tissue Res (2016) 365(3):553–62. 10.1007/s00441-016-2461-3 27423661

[34] Ye LY Zhang Q Bai XL Pankaj P Hu QD Liang TB. Hypoxia-Inducible Factor 1alpha Expression and its Clinical Significance in Pancreatic Cancer: A Meta-Analysis. Pancreatology (2014) 14(5):391–7. 10.1016/j.pan.2014.06.008 25278309

[35] Shen W Li HL Liu L Cheng JX. Expression Levels of PTEN, HIF-1alpha, and VEGF as Prognostic Factors in Ovarian Cancer. Eur Rev Med Pharmacol Sci (2017) 21(11):2596–603. 28678326

[36] Gonzalez H Hagerling C Werb Z. Roles of the Immune System in Cancer: From Tumor Initiation to Metastatic Progression. Genes Dev (2018) 32(19-20):1267–84. 10.1101/gad.314617.118 PMC6169832 30275043

[37] Alazawi W Pirmadjid N Lahiri R Bhattacharya S. Inflammatory and Immune Responses to Surgery and Their Clinical Impact. Ann Surg (2016) 264(1):73–80. 10.1097/SLA.0000000000001691 27275778

[38] Kurosawa S Kato M. Anesthetics, Immune Cells, and Immune Responses. J Anesth (2008) 22(3):263–77. 10.1007/s00540-008-0626-2 18685933

[39] Angka L Khan ST Kilgour MK Xu R Kennedy MA Auer RC. Dysfunctional Natural Killer Cells in the Aftermath of Cancer Surgery. Int J Mol Sci (2017) 18(8). 10.3390/ijms18081787 PMC5578175 28817109

[40] Decker D Schondorf M Bidlingmaier F Hirner A von Ruecker AA. Surgical Stress Induces a Shift in the Type-1/Type-2 T-Helper Cell Balance, Suggesting Down-Regulation of Cell-Mediated and Up-Regulation of Antibody-Mediated Immunity Commensurate to the Trauma. Surgery (1996) 119(3):316–25. 10.1016/S0039-6060(96)80118-8 8619187

[41] Hsu BE Shen Y Siegel PM. Neutrophils: Orchestrators of the Malignant Phenotype. Front Immunol (2020) 11. 10.3389/fimmu.2020.01778 PMC7433712 32849639

[42] Howard R Kanetsky PA Egan KM. Exploring the Prognostic Value of the Neutrophil-to-Lymphocyte Ratio in Cancer. Sci Rep (2019) 9(1):19673. 10.1038/s41598-019-56218-z 31873162 PMC6928022

[43] Templeton AJ McNamara MG Šeruga B Vera-Badillo FE Aneja P Ocaña A. Prognostic Role of Neutrophil-to-Lymphocyte Ratio in Solid Tumors: A Systematic Review and Meta-Analysis. J Natl Cancer Inst (2014) 106(6):dju124. 10.1093/jnci/dju124 24875653

[44] Fridlender ZG Sun J Kim S Kapoor V Cheng G Ling L. Polarization of Tumor-Associated Neutrophil Phenotype by TGF-Beta: "N1" Versus "N2" TAN. Cancer Cell (2009) 16(3):183–94. 10.1016/j.ccr.2009.06.017 PMC2754404 19732719

[45] Liang W Ferrara N. The Complex Role of Neutrophils in Tumor Angiogenesis and Metastasis. Cancer Immunol Res (2016) 4(2):83–91. 10.1158/2326-6066.CIR-15-0313 26839309

[46] Masucci MT Minopoli M Del Vecchio S Carriero MV. The Emerging Role of Neutrophil Extracellular Traps (NETs) in Tumor Progression and Metastasis. Front Immunol (2020) 11:1749. 10.3389/fimmu.2020.01749 33042107 PMC7524869

[47] Tohme S Yazdani HO Al-Khafaji AB Chidi AP Loughran P Mowen K. Neutrophil Extracellular Traps Promote the Development and Progression of Liver Metastases After Surgical Stress. Cancer Res (2016) 76(6):1367–80. 10.1158/0008-5472.CAN-15-1591 PMC4794393 26759232

[48] Grilz E Mauracher LM Posch F Königsbrügge O Zöchbauer-Müller S Marosi C. Citrullinated Histone H3, a Biomarker for Neutrophil Extracellular Trap Formation, Predicts the Risk of Mortality in Patients With Cancer. Br J Haematol (2019) 186(2):311–20. 10.1111/bjh.15906 PMC6618331 30968400

[49] Cools-Lartigue J Spicer J McDonald B Gowing S Chow S Giannias B. Neutrophil Extracellular Traps Sequester Circulating Tumor Cells and Promote Metastasis. J Clin Invest (2013) 123(8):3446–58. 10.1172/JCI67484 PMC3726160 23863628

[50] Teijeira Á Garasa S Gato M Alfaro C Migueliz I Cirella A. CXCR1 and CXCR2 Chemokine Receptor Agonists Produced by Tumors Induce Neutrophil Extracellular Traps That Interfere With Immune Cytotoxicity. Immunity (2020) 52(5):856–71.e8. 10.1016/j.immuni.2020.03.001 32289253

[51] Chlebowski RT Block JB Cundiff D Dietrich MF. Doxorubicin Cytotoxicity Enhanced by Local Anesthetics in a Human Melanoma Cell Line. Cancer Treat Rep (1982) 66(1):121–5. 7053248

[52] Grandhi RK Perona B. Mechanisms of Action by Which Local Anesthetics Reduce Cancer Recurrence: A Systematic Review. Pain Med (2020) 21(2):401–14. 10.1093/pm/pnz139 31282958

[53] D'Agostino G Saporito A Cecchinato V Silvestri Y Borgeat A Anselmi L. Lidocaine Inhibits Cytoskeletal Remodelling and Human Breast Cancer Cell Migration. Br J Anaesth (2018) 121(4):962–8. 10.1016/j.bja.2018.07.015 30236259

[54] Li R Xiao C Liu H Huang Y Dilger JP Lin J. Effects of Local Anesthetics on Breast Cancer Cell Viability and Migration. BMC Cancer (2018) 18(1):666. 10.1186/s12885-018-4576-2 29914426 PMC6006780

[55] Zhu J Han S. Lidocaine Inhibits Cervical Cancer Cell Proliferation and Induces Cell Apoptosis by Modulating the lncRNA-MEG3/miR-421/BTG1 Pathway. Am J Transl Res (2019) 11(9):5404–16. PMC6789266 31632519

[56] Zhang X Pang W Liu H Wang J. Lidocine Potentiates the Cytotoxicity of 5-Fluorouracil to Choriocarcinoma Cells by Downregulating ABC Transport Proteins Expression. J Cell Biochem (2019) 120(10):16533–42. 10.1002/jcb.28913 31081972

[57] Qu X Yang L Shi Q Wang X Wang D Wu G. Lidocaine Inhibits Proliferation and Induces Apoptosis in Colorectal Cancer Cells by Upregulating Mir-520a-3p and Targeting EGFR. Pathol Res Pract (2018) 214(12):1974–9. 10.1016/j.prp.2018.09.012 30262429

[58] Siekmann W Tina E Von Sydow AK Gupta A. Effect of Lidocaine and Ropivacaine on Primary (SW480) and Metastatic (SW620) Colon Cancer Cell Lines. Oncol Lett (2019) 18(1):395–401. 10.3892/ol.2019.10332 31497075 PMC6728124

[59] Tat T Jurj A Selicean C Pasca S Ionescu D. Antiproliferative Effects of Propofol and Lidocaine on the Colon Adenocarcinoma Microenvironment. J buon (2019) 24(1):106–15. 30941958

[60] Bundscherer AC Malsy M Bitzinger DI Wiese CH Gruber MA Graf BM. Effects of Lidocaine on HT-29 and SW480 Colon Cancer Cells In Vitro. Anticancer Res (2017) 37(4):1941–5. 10.21873/anticanres.11534 28373464

[61] Zhu G Zhang L Dan J Zhu Q. Differential Effects and Mechanisms of Local Anesthetics on Esophageal Carcinoma Cell Migration, Growth, Survival and Chemosensitivity. BMC Anesthesiol (2020) 20(1):126. 10.1186/s12871-020-01039-1 32450791 PMC7249391

[62] Ye L Zhang Y Chen YJ Liu Q. Anti-Tumor Effects of Lidocaine on Human Gastric Cancer Cells In Vitro. Bratisl Lek Listy (2019) 120(3):212–7. 10.4149/BLL_2019_036 31023040

[63] Sui H Lou A Li Z Yang J. Lidocaine Inhibits Growth, Migration and Invasion of Gastric Carcinoma Cells by Up-Regulation of miR-145. BMC Cancer (2019) 19(1):233. 10.1186/s12885-019-5431-9 30876463 PMC6419442

[64] Yang W Cai J Zhang H Wang G Jiang W. Effects of Lidocaine and Ropivacaine on Gastric Cancer Cells Through Down-Regulation of ERK1/2 Phosphorylation In Vitro. Anticancer Res (2018) 38(12):6729–35. 10.21873/anticanres.13042 30504383

[65] Zhang X Gu G Li X Zhang C. Lidocaine Alleviates Cisplatin Resistance and Inhibits Migration of MGC-803/DDP Cells Through Decreasing miR-10b. Cell Cycle (2020) 19(19):2530–7. 10.1080/15384101.2020.1809914 PMC7553558 32892697

[66] Izdebska M Hałas-Wiśniewska M Zielińska W Klimaszewska-Wiśniewska A Grzanka D Gagat M. Lidocaine Induces Protective Autophagy in Rat C6 Glioma Cell Line. Int J Oncol (2019) 54(3):1099–111. 10.3892/ijo.2018.4668 PMC6365045 30569147

[67] Leng T Lin S Xiong Z Lin J. Lidocaine Suppresses Glioma Cell Proliferation by Inhibiting TRPM7 Channels. Int J Physiol Pathophysiol Pharmacol (2017) 9(2):8–15. 28533887 PMC5435668

[68] Liu H Wang Y Chen B Shen X Li W. Effects of Lidocaine-Mediated CPEB3 Upregulation in Human Hepatocellular Carcinoma Cell Proliferation In Vitro. BioMed Res Int (2018) 2018:8403157. 10.1155/2018/8403157 29850575 PMC5932519

[69] Jurj A Tomuleasa C Tat TT Berindan-Neagoe I Vesa SV Ionescu DC. Antiproliferative and Apoptotic Effects of Lidocaine on Human Hepatocarcinoma Cells. A Preliminary Study. J Gastrointestin Liver Dis (2017) 26(1):45–50. 10.15403/jgld.2014.1121.261.juj 28338113

[70] Le Gac G Angenard G Clement B Laviolle B Coulouarn C Beloeil H. Local Anesthetics Inhibit the Growth of Human Hepatocellular Carcinoma Cells. Anesth Analg (2017) 125(5):1600–9. 10.1213/ANE.0000000000002429 28857796

[71] Ni J Xie T Xiao M Xiang W Wang L. Amide-Linked Local Anesthetics Preferentially Target Leukemia Stem Cell Through Inhibition of Wnt/β-Catenin. Biochem Biophys Res Commun (2018) 503(2):956–62. 10.1016/j.bbrc.2018.06.102 29932919

[72] Sun H Sun Y. Lidocaine Inhibits Proliferation and Metastasis of Lung Cancer Cell via Regulation of miR-539/EGFR Axis. Artif Cells Nanomed Biotechnol (2019) 47(1):2866–74. 10.1080/21691401.2019.1636807 31299862

[73] Zhang L Hu R Cheng Y Wu X Xi S Sun Y. Lidocaine Inhibits the Proliferation of Lung Cancer by Regulating the Expression of GOLT1A. Cell Prolif (2017) 50(5). 10.1111/cpr.12364 PMC6529060 28737263

[74] Yang Q Zhang Z Xu H Ma C. Lidocaine Alleviates Cytotoxicity-Resistance in Lung Cancer A549/DDP Cells via Down-Regulation of miR-21. Mol Cell Biochem (2019) 456(1-2):63–72. 10.1007/s11010-018-3490-x 30644017

[75] Piegeler T Schlapfer M Dull RO Schwartz DE Borgeat A Minshall RD. Clinically Relevant Concentrations of Lidocaine and Ropivacaine Inhibit TNFalpha-Induced Invasion of Lung Adenocarcinoma Cells In Vitro by Blocking the Activation of Akt and Focal Adhesion Kinase. Br J Anaesth (2015) 115(5):784–91. 10.1093/bja/aev341 PMC4850926 26475807

[76] Dong Q Mao Z. The Local Anaesthetic Lignocaine Exhibits Potent Antilung Cancer Cell Activity by Inhibiting the Phosphoinositide 3-Kinases/Mammalian Target of Rapamycin/Mammalian Target of Rapamycin Pathway. Pharmacology (2019) 104(3-4):139–46. 10.1159/000500743 31203272

[77] Wang HW Wang LY Jiang L Tian SM Zhong TD Fang XM. Amide-Linked Local Anesthetics Induce Apoptosis in Human Non-Small Cell Lung Cancer. J Thorac Dis (2016) 8(10):2748–57. 10.21037/jtd.2016.09.66 PMC5107467 27867550

[78] Zheng Q Peng X Zhang Y. Cytotoxicity of Amide-Linked Local Anesthetics on Melanoma Cells via Inhibition of Ras and RhoA Signaling Independent of Sodium Channel Blockade. BMC Anesthesiol (2020) 20(1):43. 10.1186/s12871-020-00957-4 32085741 PMC7033929

[79] Wang Y Xie J Liu W Zhang R Huang S Xing Y. Lidocaine Sensitizes the Cytotoxicity of 5-Fluorouacil in Melanoma Cells via Upregulation of microRNA-493. Pharmazie (2017) 72(11):663–9. 10.1691/ph.2017.7616 29442040

[80] Mirshahidi S Shields TG de Necochea-Campion R Yuan X Janjua A Williams NL. Bupivacaine and Lidocaine Induce Apoptosis in Osteosarcoma Tumor Cells. Clin Orthop Relat Res (2021) 479(1):180–94. 10.1097/CORR.0000000000001510 PMC7899706 33009230

[81] Chang YC Hsu YC Liu CL Huang SY Hu MC Cheng SP. Local Anesthetics Induce Apoptosis in Human Thyroid Cancer Cells Through the Mitogen-Activated Protein Kinase Pathway. PLoS One (2014) 9(2):e89563. 10.1371/journal.pone.0089563 24586874 PMC3931808

[82] Cassuto J Sinclair R Bonderovic M. Anti-Inflammatory Properties of Local Anesthetics and Their Present and Potential Clinical Implications. Acta Anaesthesiol Scand (2006) 50(3):265–82. 10.1111/j.1399-6576.2006.00936.x 16480459

[83] Saeidnia S Manayi A Abdollahi M. From In Vitro Experiments to In Vivo and Clinical Studies; Pros and Cons. Curr Drug Discov Technol (2015) 12(4):218–24. 10.2174/1570163813666160114093140 26778084

[84] Chamaraux-Tran TN Mathelin C Aprahamian M Joshi GP Tomasetto C Diemunsch P. Antitumor Effects of Lidocaine on Human Breast Cancer Cells: An In Vitro and In Vivo Experimental Trial. Anticancer Res (2018) 38(1):95–105. 10.21873/anticanres.12196 29277761

[85] Yang X Zhao L Li M Yan L Zhang S Mi Z. Lidocaine Enhances the Effects of Chemotherapeutic Drugs Against Bladder Cancer. Sci Rep (2018) 8(1):598. 10.1038/s41598-017-19026-x 29330444 PMC5766619

[86] Wall TP Crowley PD Sherwin A Foley AG Buggy DJ. Effects of Lidocaine and Src Inhibition on Metastasis in a Murine Model of Breast Cancer Surgery. Cancers (Basel) (2019) 11(10). 10.3390/cancers11101414 PMC6826872 31546727

[87] Johnson MZ Crowley PD Foley AG Xue C Connolly C Gallagher HC. Effect of Perioperative Lidocaine on Metastasis After Sevoflurane or Ketamine-Xylazine Anaesthesia for Breast Tumour Resection in a Murine Model. Br J Anaesth (2018) 121(1):76–85. 10.1016/j.bja.2017.12.043 29935598

[88] Freeman J Crowley PD Foley AG Gallagher HC Iwasaki M Ma D. Effect of Perioperative Lidocaine and Cisplatin on Metastasis in a Murine Model of Breast Cancer Surgery. Anticancer Res (2018) 38(10):5599–606. 10.21873/anticanres.12894 30275177

[89] Freeman J Crowley PD Foley AG Gallagher HC Iwasaki M Ma D. Effect of Perioperative Lidocaine, Propofol and Steroids on Pulmonary Metastasis in a Murine Model of Breast Cancer Surgery. Cancers (Basel) (2019) 11(5). 10.3390/cancers11050613 PMC6562941 31052479

[90] Chen J Jiao Z Wang A Zhong W. Lidocaine Inhibits Melanoma Cell Proliferation by Regulating ERK Phosphorylation. J Cell Biochem (2019) 120(4):6402–8. 10.1002/jcb.27927 30430626

[91] Gao J Hu H Wang X. Clinically Relevant Concentrations of Lidocaine Inhibit Tumor Angiogenesis Through Suppressing VEGF/VEGFR2 Signaling. Cancer Chemother Pharmacol (2019) 83(6):1007–15. 10.1007/s00280-019-03815-4 30887179

[92] Xia W Wang L Yu D Mu X Zhou X. Lidocaine Inhibits the Progression of Retinoblastoma In Vitro and In Vivo by Modulating the Mir−520a−3p/EGFR Axis. Mol Med Rep (2019) 20(2):1333–42. 10.3892/mmr.2019.10363 PMC6625385 31173241

[93] Xing W Chen DT Pan JH Chen YH Yan Y Li Q. Lidocaine Induces Apoptosis and Suppresses Tumor Growth in Human Hepatocellular Carcinoma Cells In Vitro and in a Xenograft Model In Vivo. Anesthesiology (2017) 126(5):868–81. 10.1097/ALN.0000000000001528 28121635

[94] Edlich F. BCL-2 Proteins and Apoptosis: Recent Insights and Unknowns. Biochem Biophys Res Commun (2018) 500(1):26–34. 10.1016/j.bbrc.2017.06.190 28676391

[95] Van Opdenbosch N Lamkanfi M. Caspases in Cell Death, Inflammation, and Disease. Immunity (2019) 50(6):1352–64. 10.1016/j.immuni.2019.05.020 PMC6611727 31216460

[96] Youle RJ Strasser A. The BCL-2 Protein Family: Opposing Activities That Mediate Cell Death. Nat Rev Mol Cell Biol (2008) 9(1):47–59. 10.1038/nrm2308 18097445

[97] Papa S Choy PM Bubici C. The ERK and JNK Pathways in the Regulation of Metabolic Reprogramming. Oncogene (2019) 38(13):2223–40. 10.1038/s41388-018-0582-8 PMC6398583 30487597

[98] Yue J López JM. Understanding MAPK Signaling Pathways in Apoptosis. Int J Mol Sci (2020) 21(7). 10.3390/ijms21072346 PMC7177758 32231094

[99] Roskoski R Jr. Small Molecule Inhibitors Targeting the EGFR/ErbB Family of Protein-Tyrosine Kinases in Human Cancers. Pharmacol Res (2019) 139:395–411. 10.1016/j.phrs.2018.11.014 30500458

[100] Hoesel B Schmid JA. The Complexity of NF-κb Signaling in Inflammation and Cancer. Mol Cancer (2013) 12:86. 10.1186/1476-4598-12-86 23915189 PMC3750319

[101] Zhang Q Lenardo MJ Baltimore D. 30 Years of NF-κb: A Blossoming of Relevance to Human Pathobiology. Cell (2017) 168(1-2):37–57. 10.1016/j.cell.2016.12.012 28086098 PMC5268070

[102] Wang HL Xing YQ Xu YX Rong F Lei WF Zhang WH. The Protective Effect of Lidocaine on Septic Rats via the Inhibition of High Mobility Group Box 1 Expression and NF-κb Activation. Mediators Inflamm (2013) 2013:570370. 10.1155/2013/570370 24371375 PMC3858876

[103] Sirait RH Hatta M Ramli M Islam AA Arief SK. Systemic Lidocaine Inhibits High-Mobility Group Box 1 Messenger Ribonucleic Acid Expression and Protein in BALB/c Mice After Closed Fracture Musculoskeletal Injury. Saudi J Anaesth (2018) 12(3):395–8. 10.4103/sja.SJA_685_17 PMC6044174 30100837

[104] Wang HL Liu YY Yan HD Wang XS Huang R Lei WF. Intraoperative Systemic Lidocaine Inhibits the Expression of HMGB1 in Patients Undergoing Radical Hysterectomy. Int J Clin Exp Med (2014) 7(10):3398–403. PMC4238473 25419374

[105] Lahat A Ben-Horin S Lang A Fudim E Picard O Chowers Y. Lidocaine Down-Regulates Nuclear factor-kappaB Signalling and Inhibits Cytokine Production and T Cell Proliferation. Clin Exp Immunol (2008) 152(2):320–7. 10.1111/j.1365-2249.2008.03636.x PMC2384107 18355353

[106] Komiya Y Habas R. Wnt Signal Transduction Pathways. Organogenesis (2008) 4(2):68–75. 10.4161/org.4.2.5851 19279717 PMC2634250

[107] Jung Y-S Park J-I. Wnt Signaling in Cancer: Therapeutic Targeting of Wnt Signaling Beyond β-Catenin and the Destruction Complex. Exp Mol Med (2020) 52(2):183–91. 10.1038/s12276-020-0380-6 PMC7062731 32037398

[108] Stamos JL Weis WI. The β-Catenin Destruction Complex. Cold Spring Harb Perspect Biol (2013) 5(1):a007898. 10.1101/cshperspect.a007898 23169527 PMC3579403

[109] Fels B Bulk E Pethő Z Schwab A. The Role of TRP Channels in the Metastatic Cascade. Pharmaceuticals (Basel) (2018) 11(2). 10.3390/ph11020048 PMC6027473 29772843

[110] Zhou W Guo S Xiong Z Liu M. Oncogenic Role and Therapeutic Target of Transient Receptor Potential Melastatin 7 Channel in Malignancy. Expert Opin Ther Targets (2014) 18(10):1177–96. 10.1517/14728222.2014.940894 25069584

[111] Leng TD Lin J Sun HW Zeng Z O'Bryant Z Inoue K. Local Anesthetic Lidocaine Inhibits TRPM7 Current and TRPM7-Mediated Zinc Toxicity. CNS Neurosci Ther (2015) 21(1):32–9. 10.1111/cns.12318 PMC6495118 25169754

[112] Leng TD Li MH Shen JF Liu ML Li XB Sun HW. Suppression of TRPM7 Inhibits Proliferation, Migration, and Invasion of Malignant Human Glioma Cells. CNS Neurosci Ther (2015) 21(3):252–61. 10.1111/cns.12354 PMC4339490 25438992

[113] Liu H Dilger JP Lin J. Lidocaine Suppresses Viability and Migration of Human Breast Cancer Cells: TRPM7 as a Target for Some Breast Cancer Cell Lines. Cancers (Basel) (2021) 13(2). 10.3390/cancers13020234 PMC7827240 33435261

[114] Jiang Y Gou H Zhu J Tian S Yu L. Lidocaine Inhibits the Invasion and Migration of TRPV6-Expressing Cancer Cells by TRPV6 Downregulation. Oncol Lett (2016) 12(2):1164–70. 10.3892/ol.2016.4709 PMC4950910 27446413

[115] Lu J Ju Y-T Li C Hua F-Z Xu G-H Hu Y-H. Effect of TRPV1 Combined With Lidocaine on Cell State and Apoptosis of U87-MG Glioma Cell Lines. Asian Pacific J Trop Med (2016) 9(3):288–92. 10.1016/j.apjtm.2016.01.030 26972404

[116] Irby RB Yeatman TJ. Role of Src Expression and Activation in Human Cancer. Oncogene (2000) 19(49):5636–42. 10.1038/sj.onc.1203912 11114744

[117] Tsai CL Chen WC Hsieh HL Chi PL Hsiao LD Yang CM. TNF-α Induces Matrix Metalloproteinase-9-Dependent Soluble Intercellular Adhesion Molecule-1 Release via TRAF2-Mediated MAPKs and NF-κb Activation in Osteoblast-Like MC3T3-E1 Cells. J BioMed Sci (2014) 21(1):12. 10.1186/1423-0127-21-12 24502696 PMC3926355

[118] Roskoski R Jr. Src Protein-Tyrosine Kinase Structure, Mechanism, and Small Molecule Inhibitors. Pharmacol Res (2015) 94:9–25. 10.1016/j.phrs.2015.01.003 25662515

[119] Russello SV Shore SK. Src in Human Carcinogenesis. Front Biosci (2003) 8:s1068–73. 10.2741/1138 12957809

[120] Kuo L Chang HC Leu TH Maa MC Hung WC. Src Oncogene Activates MMP-2 Expression via the ERK/Sp1 Pathway. J Cell Physiol (2006) 207(3):729–34. 10.1002/jcp.20616 16453304

[121] Piegeler T Votta-Velis EG Liu G Place AT Schwartz DE Beck-Schimmer B. Antimetastatic Potential of Amide-Linked Local Anesthetics: Inhibition of Lung Adenocarcinoma Cell Migration and Inflammatory Src Signaling Independent of Sodium Channel Blockade. Anesthesiology (2012) 117(3):548–59. 10.1097/ALN.0b013e3182661977 PMC3482823 22846676

[122] Piegeler T Votta-Velis EG Bakhshi FR Mao M Carnegie G Bonini MG. Endothelial Barrier Protection by Local Anesthetics: Ropivacaine and Lidocaine Block Tumor Necrosis Factor-α-Induced Endothelial Cell Src Activation. Anesthesiology (2014) 120(6):1414–28. 10.1097/ALN.0000000000000174 PMC4284094 24525631

[123] Sinclair R Eriksson AS Gretzer C Cassuto J Thomsen P. Inhibitory Effects of Amide Local Anaesthetics on Stimulus-Induced Human Leukocyte Metabolic Activation, LTB4 Release and IL-1 Secretion In Vitro. Acta Anaesthesiol Scand (1993) 37(2):159–65. 10.1111/j.1399-6576.1993.tb03693.x 8383401

[124] Yanagi H Sankawa H Saito H Iikura Y. Effect of Lidocaine on Histamine Release and Ca2+ Mobilization From Mast Cells and Basophils. Acta Anaesthesiol Scand (1996) 40(9):1138–44. 10.1111/j.1399-6576.1996.tb05577.x 8933856

[125] Hollmann MW Gross A Jelacin N Durieux ME. Local Anesthetic Effects on Priming and Activation of Human Neutrophils. Anesthesiology (2001) 95(1):113–22. 10.1097/00000542-200107000-00021 11465548

[126] Waite A Gilliver SC Masterson GR Hardman MJ Ashcroft GS. Clinically Relevant Doses of Lidocaine and Bupivacaine Do Not Impair Cutaneous Wound Healing in Mice. Br J Anaesth (2010) 104(6):768–73. 10.1093/bja/aeq093 PMC2867659 20418532

[127] de Klaver MJ Buckingham MG Rich GF. Lidocaine Attenuates Cytokine-Induced Cell Injury in Endothelial and Vascular Smooth Muscle Cells. Anesth Analg (2003) 97(2):465–70. 10.1213/01.ANE.0000073162.27208.E9 12873936

[128] Ortiz MP Godoy MC Schlosser RS Ortiz RP Godoy JP Santiago ES. Effect of Endovenous Lidocaine on Analgesia and Serum Cytokines: Double-Blinded and Randomized Trial. J Clin Anesth (2016) 35:70–7. 10.1016/j.jclinane.2016.07.021 27871598

[129] Song X Sun Y Zhang X Li T Yang B. Effect of Perioperative Intravenous Lidocaine Infusion on Postoperative Recovery Following Laparoscopic Cholecystectomy-A Randomized Controlled Trial. Int J Surg (2017) 45:8–13. 10.1016/j.ijsu.2017.07.042 28705592

[130] Kuo CP Jao SW Chen KM Wong CS Yeh CC Sheen MJ. Comparison of the Effects of Thoracic Epidural Analgesia and I.V. Infusion With Lidocaine on Cytokine Response, Postoperative Pain and Bowel Function in Patients Undergoing Colonic Surgery. Br J Anaesthesia (2006) 97(5):640–6. 10.1093/bja/ael217 16952918

[131] Herroeder S Pecher S Schönherr ME Kaulitz G Hahnenkamp K Friess H. Systemic Lidocaine Shortens Length of Hospital Stay After Colorectal Surgery: A Double-Blinded, Randomized, Placebo-Controlled Trial. Ann Surg (2007) 246(2):192–200. 10.1097/SLA.0b013e31805dac11 17667496 PMC1933564

[132] Yardeni IZ Beilin B Mayburd E Levinson Y Bessler H. The Effect of Perioperative Intravenous Lidocaine on Postoperative Pain and Immune Function. Anesth Analg (2009) 109(5):1464–9. 10.1213/ANE.0b013e3181bab1bd 19843784

[133] Sridhar P Sistla SC Ali SM Karthikeyan VS Badhe AS Ananthanarayanan PH. Effect of Intravenous Lignocaine on Perioperative Stress Response and Post-Surgical Ileus in Elective Open Abdominal Surgeries: A Double-Blind Randomized Controlled Trial. ANZ J Surg (2015) 85(6):425–9. 10.1111/ans.12783 25078385

[134] Dewinter G Moens P Fieuws S Vanaudenaerde B Van de Velde M Rex S. Systemic Lidocaine Fails to Improve Postoperative Morphine Consumption, Postoperative Recovery and Quality of Life in Patients Undergoing Posterior Spinal Arthrodesis. A Double-Blind, Randomized, Placebo-Controlled Trial. Br J Anaesthesia (2017) 118(4):576–85. 10.1093/bja/aex038 28403408

[135] van den Heuvel SAS van der Wal SEI Bronkhorst EM Warlé MC Ronday M Plat J. Acute Cytokine Response During Breast Cancer Surgery: Potential Role of Dexamethasone and Lidocaine and Relationship With Postoperative Pain and Complications - Analysis of Three Pooled Pilot Randomized Controlled Trials. J Pain Res (2020) 13:1243–54. 10.2147/JPR.S252377 PMC7266394 32547185

[136] Oliveira CM Sakata RK Slullitel A Salomão R Lanchote VL Issy AM. Effect of Intraoperative Intravenous Lidocaine on Pain and Plasma Interleukin-6 in Patients Undergoing Hysterectomy. Rev Bras Anestesiol (2015) 65(2):92–8. 10.1016/j.bjane.2013.07.017 25740274

[137] Xu S Hu S Ju X Li Y Li Q Wang S. Effects of Intravenous Lidocaine, Dexmedetomidine, and Their Combination on IL-1, IL-6 and TNF-α in Patients Undergoing Laparoscopic Hysterectomy: A Prospective, Randomized Controlled Trial. BMC Anesthesiol (2021) 21(1):3. 10.1186/s12871-020-01219-z 33407156 PMC7786488

[138] Eltzschig HK Carmeliet P. Hypoxia and Inflammation. N Engl J Med (2011) 364(7):656–65. 10.1056/NEJMra0910283 PMC3930928 21323543

[139] Suzuki S Mori A Fukui A Ema Y Nishiwaki K. Lidocaine Inhibits Vascular Endothelial Growth Factor-A-Induced Angiogenesis. J Anesth (2020) 34(6):857–64. 10.1007/s00540-020-02830-7 32734387

[140] Nishi K Hirota K Takabuchi S Oda S Fukuda K Adachi T. The Effects of Local Anesthetics on Cellular Hypoxia-Induced Gene Responses Mediated by Hypoxia-Inducible Factor 1. J Anesth (2005) 19(1):54–9. 10.1007/s00540-004-0271-3 15674517

[141] Yan T Zhang GH Wang BN Sun L Zheng H. Effects of Propofol/Remifentanil-Based Total Intravenous Anesthesia Versus Sevoflurane-Based Inhalational Anesthesia on the Release of VEGF-C and TGF-Beta and Prognosis After Breast Cancer Surgery: A Prospective, Randomized and Controlled Study. BMC Anesthesiol (2018) 18(1):131. 10.1186/s12871-018-0588-3 30243294 PMC6151192

[142] Looney M Doran P Buggy DJ. Effect of Anesthetic Technique on Serum Vascular Endothelial Growth Factor C and Transforming Growth Factor β in Women Undergoing Anesthesia and Surgery for Breast Cancer. Anesthesiology (2010) 113(5):1118–25. 10.1097/ALN.0b013e3181f79a69 20930611

[143] Galoș EV Tat TF Popa R Efrimescu CI Finnerty D Buggy DJ. Neutrophil Extracellular Trapping and Angiogenesis Biomarkers After Intravenous or Inhalation Anaesthesia With or Without Intravenous Lidocaine for Breast Cancer Surgery: A Prospective, Randomised Trial. Br J Anaesth (2020) 125(5):712–21. 10.1016/j.bja.2020.05.003 32616309

[144] El-Tahan MR Warda OM Diab DG Ramzy EA Matter MK. A Randomized Study of the Effects of Perioperative I.V. Lidocaine on Hemodynamic and Hormonal Responses for Cesarean Section. J Anesth (2009) 23(2):215–21. 10.1007/s00540-009-0738-3 19444560

[145] Wallin G Cassuto J Högström S Lindén I Faxén A Rimbäck G. Effects of Lidocaine Infusion on the Sympathetic Response to Abdominal Surgery. Anesth Analg (1987) 66(10):1008–13. 10.1213/00000539-198710000-00017 3631561

[146] Kaba A Laurent SR Detroz BJ Sessler DI Durieux ME Lamy ML. Intravenous Lidocaine Infusion Facilitates Acute Rehabilitation After Laparoscopic Colectomy. Anesthesiology (2007) 106(1):11–8. 10.1097/00000542-200701000-00007 17197840

[147] Birch K Jørgensen J Chraemmer-Jørgensen B Kehlet H. Effect of I.V. Lignocaine on Pain and the Endocrine Metabolic Responses After Surgery. Br J Anaesth (1987) 59(6):721–4. 10.1093/bja/59.6.721 3606915

[148] Jeon YT Na H Ryu H Chung Y. Modulation of Dendritic Cell Activation and Subsequent Th1 Cell Polarization by Lidocaine. PLoS One (2015) 10(10):e0139845. 10.1371/journal.pone.0139845 26445366 PMC4596553

[149] Gray A Marrero-Berrios I Weinberg J Manchikalapati D SchianodiCola J Schloss RS. The Effect of Local Anesthetic on Pro-Inflammatory Macrophage Modulation by Mesenchymal Stromal Cells. Int Immunopharmacol (2016) 33:48–54. 10.1016/j.intimp.2016.01.019 26854576 PMC4779686

[150] Ramirez MF Tran P Cata JP. The Effect of Clinically Therapeutic Plasma Concentrations of Lidocaine on Natural Killer Cell Cytotoxicity. Reg Anesth Pain Med (2015) 40(1):43–8. 10.1097/AAP.0000000000000191 25469757

[151] Cata JP Ramirez MF Velasquez JF Di AI Popat KU Gottumukkala V. Lidocaine Stimulates the Function of Natural Killer Cells in Different Experimental Settings. Anticancer Res (2017) 37(9):4727–32. 10.21873/anticanres.11879 28870891

[152] Wang HL Yan HD Liu YY Sun BZ Huang R Wang XS. Intraoperative Intravenous Lidocaine Exerts a Protective Effect on Cell-Mediated Immunity in Patients Undergoing Radical Hysterectomy. Mol Med Rep (2015) 12(5):7039–44. 10.3892/mmr.2015.4235 26299324

[153] Yokoyama M Itano Y Mizobuchi S Nakatsuka H Kaku R Takashima T. The Effects of Epidural Block on the Distribution of Lymphocyte Subsets and Natural-Killer Cell Activity in Patients With and Without Pain. Anesth Analg (2001) 92(2):463–9. 10.1213/00000539-200102000-00035 11159252

[154] Lan W Harmon D Wang JH Shorten G Redmond P. The Effect of Lidocaine on Neutrophil CD11b/CD18 and Endothelial ICAM-1 Expression and IL-1beta Concentrations Induced by Hypoxia-Reoxygenation. Eur J Anaesthesiol (2004) 21(12):967–72. 10.1017/S0265021504000353 15719860

[155] Poffers M Bühne N Herzog C Thorenz A Chen R Güler F. Sodium Channel Nav1.3 Is Expressed by Polymorphonuclear Neutrophils During Mouse Heart and Kidney Ischemia InVivo and Regulates Adhesion, Transmigration, and Chemotaxis of Human and Mouse Neutrophils In Vitro. Anesthesiology (2018) 128(6):1151–66. 10.1097/ALN.0000000000002135 29509584

[156] Chiang N Schwab JM Fredman G Kasuga K Gelman S Serhan CN. Anesthetics Impact the Resolution of Inflammation. PLoS One (2008) 3(4):e1879. 10.1371/journal.pone.0001879 18382663 PMC2268966

[157] MacGregor RR Thorner RE Wright DM. Lidocaine Inhibits Granulocyte Adherence and Prevents Granulocyte Delivery to Inflammatory Sites. Blood (1980) 56(2):203–9. 10.1182/blood.V56.2.203.203 7397378

[158] Scott BD Shasby DM Tomanek RJ Kieso RA Seabold JE Ponto JA. Lidocaine and Dextran Sulfate Inhibit Leukocyte Accumulation But Not Postischemic Contractile Dysfunction in a Canine Model. Am Heart J (1993) 125(4):1002–11. 10.1016/0002-8703(93)90107-K 8465722

[159] Berger C Rossaint J Van Aken H Westphal M Hahnenkamp K Zarbock A. Lidocaine Reduces Neutrophil Recruitment by Abolishing Chemokine-Induced Arrest and Transendothelial Migration in Septic Patients. J Immunol (2014) 192(1):367–76. 10.4049/jimmunol.1301363 24293632

[160] Hyvönen PM Kowolik MJ. Dose-Dependent Suppression of the Neutrophil Respiratory Burst by Lidocaine. Acta Anaesthesiol Scand (1998) 42(5):565–9. 10.1111/j.1399-6576.1998.tb05167.x 9605373

[161] Kawasaki C Kawasaki T Ogata M Sata T Chaudry IH. Lidocaine Enhances Apoptosis and Suppresses Mitochondrial Functions of Human Neutrophil In Vitro. J Trauma (2010) 68(2):401–8. 10.1097/TA.0b013e3181af6e56 19996799

[162] Billert H Czerniak K Bednarek E Kulińska K. Effects of Local Anesthetics on the Respiratory Burst of Cord Blood Neutrophils In Vitro. Pediatr Res (2016) 80(2):258–66. 10.1038/pr.2016.68 27055189

[163] Kiefer RT Ploppa A Krueger WA Plank M Nohé B Haeberle HA. Local Anesthetics Impair Human Granulocyte Phagocytosis Activity, Oxidative Burst, and CD11b Expression in Response to Staphylococcus Aureus. Anesthesiology (2003) 98(4):842–8. 10.1097/00000542-200304000-00009 12657844

[164] Ploppa A Kiefer RT Krueger WA Unertl KE Durieux ME. Local Anesthetics Time-Dependently Inhibit Staphylococcus Aureus Phagocytosis, Oxidative Burst and CD11b Expression by Human Neutrophils. Reg Anesth Pain Med (2008) 33(4):297–303. 10.1097/00115550-200807000-00003 18675739

[165] Mikawa K Akamarsu H Nishina K Shiga M Obara H Niwa Y. Effects of Ropivacaine on Human Neutrophil Function: Comparison With Bupivacaine and Lidocaine. Eur J Anaesthesiol (2003) 20(2):104–10. 10.1097/00003643-200302000-00004 12622492

[166] Peck SL Johnston RB Jr. Horwitz LD. Reduced Neutrophil Superoxide Anion Release After Prolonged Infusions of Lidocaine. J Pharmacol Exp Ther (1985) 235(2):418–22. 2997433

[167] Swanton BJ Iohom G Wang JH Redmond HP Shorten GD. The Effect of Lidocaine on Neutrophil Respiratory Burst During Induction of General Anaesthesia and Tracheal Intubation. Eur J Anaesthesiol (2001) 18(8):524–9. 10.1097/00003643-200108000-00007 11473559

[168] Ni Eochagain A Burns D Riedel B Sessler DI Buggy DJ. The Effect of Anaesthetic Technique During Primary Breast Cancer Surgery on Neutrophil-Lymphocyte Ratio, Platelet-Lymphocyte Ratio and Return to Intended Oncological Therapy. Anaesthesia (2018) 73(5):603–11. 10.1111/anae.14207 29457215

[169] Surhonne N Hebri C Kannan S Duggappa DR Rs RR Mapari CG. The Effect of Anesthetic Techniques on Neutrophil to Lymphocyte Ratio in Patients Undergoing Infraumbilical Surgeries. Korean J Anesthesiol (2019) 72(5):458–65. 10.4097/kja.d.19.00022 PMC6781208 31096728

[170] Memary E Mirkheshti A Ghasemi M Taheri M Arhami Dolatabadi A Kaboudvand A. The Effect of Lidocaine Infusion During General Anesthesia on Neutrophil-Lymphocyte-Ratio in Breast Cancer Patients Candidate for Mastectomy; a Clinical Trial. J Cell Mol Anesthesia (2016) 1(4):146–53.

[171] Biki B Mascha E Moriarty DC Fitzpatrick JM Sessler DI Buggy DJ. Anesthetic Technique for Radical Prostatectomy Surgery Affects Cancer Recurrence: A Retrospective Analysis. Anesthesiology (2008) 109(2):180–7. 10.1097/ALN.0b013e31817f5b73 18648226

[172] Exadaktylos AK Buggy DJ Moriarty DC Mascha E Sessler DI. Can Anesthetic Technique for Primary Breast Cancer Surgery Affect Recurrence or Metastasis? Anesthesiology (2006) 105(4):660–4. 10.1097/00000542-200610000-00008 PMC1615712 17006061

[173] Sessler DI Pei L Huang Y Fleischmann E Marhofer P Kurz A. Recurrence of Breast Cancer After Regional or General Anaesthesia: A Randomised Controlled Trial. Lancet (2019) 394(10211):1807–15. 10.1016/S0140-6736(19)32313-X 31645288

[174] Zhang H Yang L Zhu X Zhu M Sun Z Cata JP. Association Between Intraoperative Intravenous Lidocaine Infusion and Survival in Patients Undergoing Pancreatectomy for Pancreatic Cancer: A Retrospective Study. Br J Anaesth (2020) 125(2):141–8. 10.1016/j.bja.2020.03.034 32475684

[175] Riedel B. Volatile Anaesthesia and Perioperative Outcomes Related to Cancer (VAPOR-C): A Feasibility Study. Camperdown, NSW, Australia: Australian New Zealand Clinical Trials Registry (2017). Available at: https://www.anzctr.org.au/Trial/Registration/TrialReview.aspx?id=373249.

[176] Paterson H. ALLEGRO Trial 2018 . Available at: http://www.isrctn.com/ISRCTN52352431.

[177] Pandit JJ McGuire N. Unlicensed Intravenous Lidocaine for Postoperative Pain: Always a Safer 'Licence to Stop' Than to Start. Anaesthesia (2021) 76(2):156–60. 10.1111/anae.15286 33141932

[178] Macfarlane AJR Gitman M Bornstein KJ El-Boghdadly K Weinberg G. Updates in Our Understanding of Local Anaesthetic Systemic Toxicity: A Narrative Review. Anaesthesia (2021) 76(Suppl 1):27–39. 10.1111/anae.15282 33426662

[179] Pandit JJ McGuire N. Intravenous Lidocaine: Benefits Require Better Evidence, and Potential Risks Apply to All Team Members. Anaesthesia (2021) 76(5):718–9. 10.1111/anae.15439 33591568

